# Feature Extraction and Machine Learning for the Classification of Brazilian Savannah Pollen Grains

**DOI:** 10.1371/journal.pone.0157044

**Published:** 2016-06-08

**Authors:** Ariadne Barbosa Gonçalves, Junior Silva Souza, Gercina Gonçalves da Silva, Marney Pascoli Cereda, Arnildo Pott, Marco Hiroshi Naka, Hemerson Pistori

**Affiliations:** 1 Department of Biotechnology, INOVISAO, Dom Bosco Catholic University, Campo Grande, Mato Grosso do Sul, Brazil; 2 Department of Computing Science, Universidade Federal de Mato Grosso do Sul, Campo Grande, Mato Grosso do Sul, Brazil; 3 Department of Environmental Science and Agricultural Sustainability, Dom Bosco Catholic University, Campo Grande, Mato Grosso do Sul, Brazil; 4 Laboratory of Botany, Universidade Federal de Mato Grosso do Sul, Campo Grande, Mato Grosso do Sul, Brazil; 5 Direction of Research, Extension and Institutional, Federal Institute of Mato Grosso do Sul, Science and Technology. Campo Grande, Mato Grosso do Sul, Brazil; University of Ulm, GERMANY

## Abstract

The classification of pollen species and types is an important task in many areas like forensic palynology, archaeological palynology and melissopalynology. This paper presents the first annotated image dataset for the Brazilian Savannah pollen types that can be used to train and test computer vision based automatic pollen classifiers. A first baseline human and computer performance for this dataset has been established using 805 pollen images of 23 pollen types. In order to access the computer performance, a combination of three feature extractors and four machine learning techniques has been implemented, fine tuned and tested. The results of these tests are also presented in this paper.

## Introduction

The analysis of pollen grains can be useful in many different scenarios, as for instance, the quality control of honey based products, the collection of evidences in a crime scene to help in criminal investigations or the reconstruction of a remote paleoenvironment through fossil pollen identification. Melissopalynology, forensic palynology and paleopalynology are some of the fields where the classification of pollen grains is an important task, that is usually conducted by the visual analysis of light microscope (LM) images.

More recently, with the introduction of microscopes that can be connected to a computer to record digital images, the use of graphical softwares started to be used to enhance the images and facilitate the analysis of pollen, but this task is still mostly accomplished through human visual inspection. This inspection involves the recognition of differences in shapes, texture and other visual features from the pollen exine that can, sometimes, be very subtle and lead to classification errors by novice palynologists [1]. This paper presents some advances regarding the construction of a computer vision system to automate the classification of pollen grains.

The contribution of this paper is threefold. First, a new annotated dataset comprising 805 images from 23 pollen types collected in the Brazilian Savannah has been constructed and made publicly available to help in the development of new computer vision systems. Second, a baseline human performance on the task of classifying these 23 pollen types has been measured and analyzed and can now be used to benchmark the computer performance. Finally, three image feature extractors and four supervised machine learning techniques have been implemented and explored in order to build a computer vision system that can classify pollen images. Experiments to find the best configuration for this vision system have been conducted and the results are reported.

The three feature extractors explored in this work are the Bag of Visual Words (BOW), Color, Shape and Texture (CST), and a combination of BOW and CST that is being called CST + BOW. For machine learning, two variations of support vector machines, SMO and C-SVC, a decision tree based classifier (J48) and the k-nearest neighbors (KNN) approach have been tested. The highest Correct Classification Rate (CCR) of 64% was achieved using CST+BOW and C-SVC.

The next section presents a brief review on the state-of-art regarding the automation of the pollen classification task and is followed by the materials and methods section. The results, discussion and conclusions are then reported, and future works are finally suggested.

## Related Work

The importance of the identification of fossil pollen for the reconstruction of remote paleoenvironments is described previously [2]. The authors searched for approaches to automate the process of pollen identification through a neural network system, which is a logical programming approach that assumes connections similar to those between human neurons, using microscopic images of three plant species. Although the classification is difficult due to structural deformities and pollen clusters, these researchers achieved up to 90% efficiency in pollen classification.

The technique of analyzing shape and texture features was previously used to classify pollen from *Urticaceae* species, some of which cause respiratory allergies [3]. The investigators also emphasized the importance of a system that would be able to recognize pollen of this family, which is the most frequent family throughout the year. The system that they developed obtained an accuracy of 89% in the classification of three pollen types, which is a markedly higher accuracy than achieved by a palynologist in routine analysis. In a research study described previously [4], the researchers reached an overall accuracy of 94% using a system that was developed based on color features to recognize the four most frequent pollen types from Spanish plants with the aim of authenticating the origin of pollen to prevent honey labeling fraud.

The development of an automatic method for pollen and honey classification with a watershed segmentation system was accomplished in a previous study [5]. These investigators used a dataset with 333 pollen images of *Fabaceae* (60 images), *Schinus* (136), *Protium* (64) and *Serjania* (73). The watershed segmentation reduced the unnecessary information in the image through a blurring and smoothing process. The best accuracy (98.9%) was found using texture entropy with a first-order histogram in a binary image.

The Bag of Visual Words technique was used to automate the recognition of nine pollen types found in honey: *Anadenanthera colubrina*, *Arecaceae*, *Cecropia pachystachya*, *Myrcia*, *Protium*, *Poaceae*, *Serjania*, *Schinus* and *Syagrus oleracea*. The technique’s performance was evaluated with five classifiers, and the best performance for all of the pollen types was obtained with SMO, which yielded a 70% accuracy in pollen classification [6].

In a previous study [7], the researchers applied color, shape and texture analysis techniques on pollen LM images for the development of a method for the automatic classification of seven pollen types found in the Midwestern region of Brazil: *A*. *colubrina*, *C*. *pachystachya*, *Myrcia*, *Protium*, *Schinus*, *Serjania* and *S*. *oleracea*. They used 30 images of each pollen type. First, the images were segmented through a watershed method. The color, shape and texture features from each image were then extracted. The Wavelet transform technique for texture extraction was used with a co-occurrence matrix of the extracted features using the second angular momentum, contrast, correlation and entropy in the images. The performance as reflected by the F-measure metric was 79%.

A database containing 345 images from 17 different pollen types has been presented in [8]. The images correspond to 17 sub-genders and species of tropical honey plants situated in Costa Rica, Central America. Using 50 image features and artificial neural networks, a Correct Classification Rate (CCR) of 92.81% has been achieved. More recently, [9] explored the use of the Bag of Visual Words technique on the classification of pollen apertures but just one pollen species, *Betula*, has been tested. They achieved 95.8% of accuracy with the best threshold parameter.

All but one of the eight papers reviewed used a significantly smaller image dataset than ours, with less than ten different pollen types. As the number of pollen types is directly related to the classification performance, the results obtained in such simpler scenarios are not comparable to ours. The largest dataset, in terms of pollen types, was presented in [8], with 17 types, still smaller than the 23 pollen types dataset presented in our work. Besides, differently from [8], we used two additional metrics to test our proposed method, F-Measure and AUC, and presented a more detailed study regarding the performance of the software on each of the pollen types using confusion matrices.

## Materials and Methods

This research had involved human participants who completed a questionnaire asking them to identify pollen samples and which pollen features they used to do pollen identification. No personal information was requested from beekeepers. This activity was previously scheduled in the beekeeping course where the questionnaire was applied. The research was verbally explained and all beekeepers were informed that it was a voluntary research. They verbally consented to participate in the research as voluntaries. This research was approved by the President of the Beekeeping and Meliponiculture Federation of Mato Grosso do Sul (FEAMS) state, in Brazil (see S1 Text).

**Fig 1:**
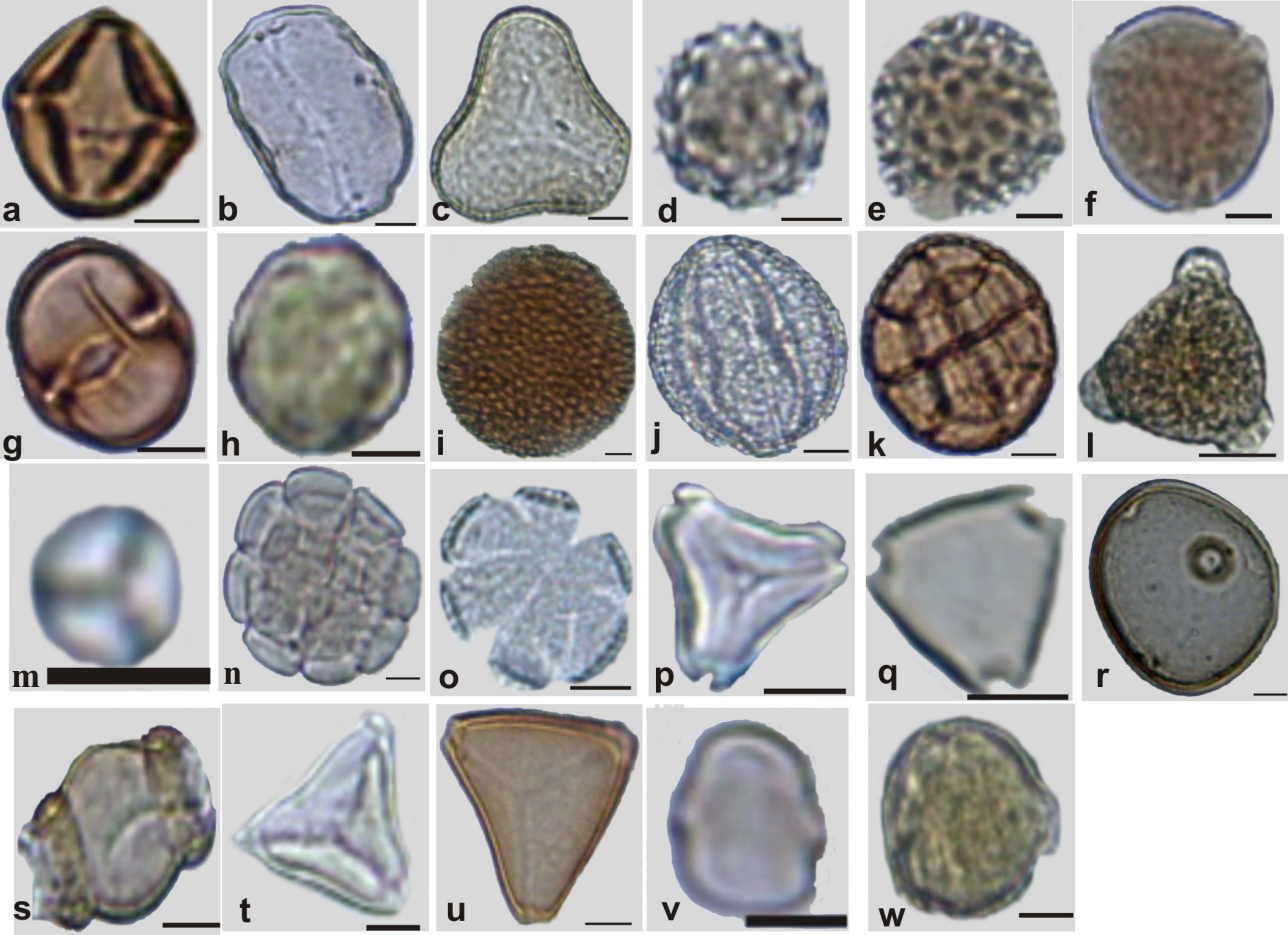
Sample images for each of the 23 pollen types from the Brazilian Savannah flora used in the experiments. **(Scale = 10 μm).** a) **Anacardiaceae:** *Schinus terebinthifolius;* b-c) **Arecaceae:** b- *Arecaceae;* c- *Syagrus*. d-e) **Asteraceae:** d) *Chromolaena laevigata;* e) *Tridax procumbens*. f) **Bignoniaceae:** *Fridericia florida*. g) **Burseraceae**: *Protium heptaphyllum*. h) **Combretaceae:** *Combretum discolor*. i-j) **Euphorbiaceae:** i) *Croton urucurana;* j) *Mabea fistulifera*. k-n) **Fabaceae:** k) *Anadenanthera colubrina;* l) *Dipteryx alata;* m) *Mimosa somnians;* n) *Senegalia plumosa;* o) **Lamiaceae:** *Hyptis*. p-q) **Myrtaceae:** p) *Eucalyptus;* q) *Myrcia*. r) **Poaceae**: *Urochloa decumbens*. s) **Rubiaceae:** *Faramea*. t-u) **Sapindaceae**: t) *Matayba guianensis;* u) *Serjania laruotteana*. v) **Urticaceae:** *Cecropia pachystachya*. w) **Vochysiaceae**: *Qualea multiflora*.

Beekeepers from the cities of the state of Mato Grosso do Sul were interviewed, and they each donated a sample of honey (1 kg) to be analyzed [10]. Through the honey analysis, it was possible to select 23 pollen types (Fig 1) in accordance with their proven importance for beekeeping and because they have been frequently found in honeys from Cerrado (Brazilian Savannah). Some of the pollen grains used in this research were reported in previous studies [5, 6, 7, 11] and are important for beekeeping in this state. The 23 pollen types used in this research were: *Anadenanthera colubrina*, *Arecaceae*, *Fridericia florida*, *Cecropia pachystachya*, *Chromolaena laevigata*, *Combretum discolor*, *Croton urucurana*, *Dipteryx alata*, *Eucalyptus*, *Faramea*, *Hyptis*, *Mabea fistulifera*, *Matayba guianensis*, *Mimosa somnians*, *Myrcia*, *Protium heptaphyllum*, *Qualea multiflora*, *Schinus terebinthifolius*, *Senegalia plumosa*, *Serjania laruotteana*, *Syagrus*, *Tridax procumbens* and *Urochloa decumbens*.

The botanist Arnildo Pott of the Federal University of Mato Grosso do Sul identified the botanical origins of the pollen grains through plant morphology identification. The *Eucalyptus* genus is not from Brazilian flora; however, in this state, there are many *Eucalyptus* plantations, which explains why eucalyptus pollen is usually found in bee products. We collected anthers from each plant and used the acetolysis method to prepare microscope pollen slides, as described previously [12].

**Fig 2:**
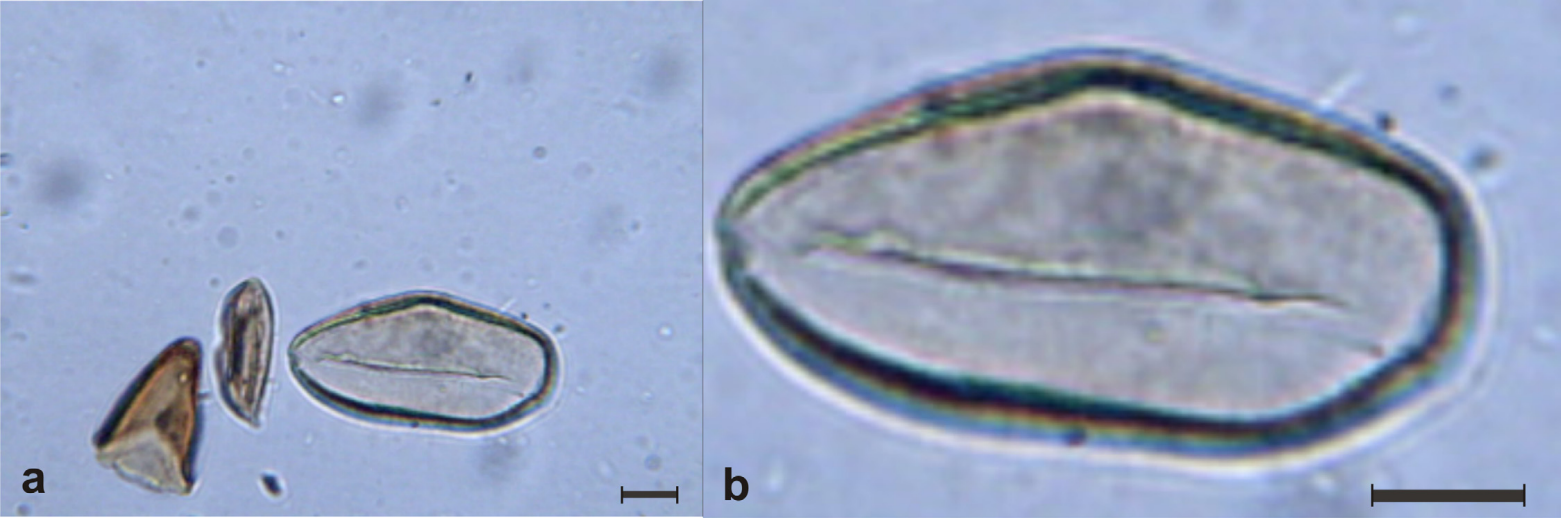
Example of the image segmentation process. a) Image captured under the light microscope and containing several pollen grains. b) Resulting segmented image containing just one pollen grain.

The dataset that comprises all of the pollen images was called POLEN23E (see S1 Dataset), where all of the pollen images were stored. This pollen dataset comprises a total of 35 images for each type of pollen taken at different angles. Thus, the POLEN23E dataset has 805 images. The images were captured with a digital Bresser LCD microscope at a 40x magnification. The best pictures were transferred to a laptop and segmented (Fig 2) using the CorelDRAW® software. The sampling method used to test the automatic classification on the POLEN23E dataset was a three fold randomized cross-validation. For the tests with humans, some of the images have been randomly chosen for training and another distinct group of images for testing.

The following subsections describe how the pollen grains were classified by human vision. Afterward, the techniques used for automatic feature extraction from the pollen images using a computer program are presented. Additionally, both the manual and automated techniques are described and evaluated using the following metrics, which are available in the Weka software system [13]: Correct Classification Rate (CCR), F-Measure and Area Under the Receiver Operating Characteristic (ROC) curve (AUC). Finally, the statistical methods that were used for the analysis of the data obtained by the techniques are reported.

### Feature Extraction Techniques

Next we describe the algorithms utilized for attribute extraction from the pollen grain images.

### Color, Shape and Texture (CST)

The extraction of features from an image is used to obtain only the relevant information that is helpful for characterization [14]. For the extraction of features from pollen images, we created an algorithm that combined three types of features: color, shape and texture based features. The algorithm created in this research that contains the mentioned features is called CST (color, shape and texture) and is referred as the CST technique.

The color-based feature is simply the arithmetic mean values on the H (hue), S (saturation), B (brightness) and R (red) color channels over the entire pollen image. The original image is coded using the standard RGB system and is converted to the HSB color-space. The G (green) and B (blue) channels are discarded based on previous studies and results regarding pollen grains classification. As regarding the shape based features, three extractors have been used: the shape factor, the circularity factor and a group of features extracted using the k-curvature algorithm [15, 16].

In the shape factor feature, the area of the pollen is divided by the area of a circumference drawn around the pollen image. The shape factor calculation is given by Eq 1

$${Area}_{object}/{Area}_{circumference}$$ (1)

where *Area_object_* corresponds to the area in number of pixels that comprise the pollen image and *Area_circumference_* corresponds to the value of the area of the minimum circumference that covers the entire image of the pollen.

The circularity factor, also called isoperimetric quotient, roughly measures how close is the pollen shape to a perfect circle. The circularity factor is given by Eq 2

$$4\;x\;{Area}_{object}/\pi \;x\;{length}^{2}$$ (2)

The k-curvature algorithm is a shape-based attribute extractor that creates a histogram that roughly counts the occurrence of curves with different angles in the outer contour of the pollen image. In this way we can have, for instance, a measure related to how many curvatures between 80 to 100 or between 100 to 120 degrees are found in the pollen shape. The parameter k is related to the length of the region around each point in the contours of the pollen image that will be used to define each curve and is determined experimentally [17].

Co-occurrence matrices, also called Gray-Level Co-occurrence Matrices (GLCM), are used to extract information related to texture [16]. In order to create a co-occurrence matrix, we calculate the frequency of pairs of intensity values from the image pixels. Different groups of pairs can be defined varying the distances and the directions or angles between the two pixels of a pair. For each distance and angle chosen, a different GLCM is created and from each GLCM the entropy and the contrast of these matrices are used as texture features. The contrast is calculated using Eq 3, i and j are indexing the columns and rows of the matrix, *n*_*g*_ is the number of different gray values (usually 255) and p(i,j) corresponds to each entry or cell of a normalized version of the GLCM [15]. Eq 4 defines the entropy feature.

$${function}_{contrast}=\overset{n_{g}-1}{\underset{n=0}{\sum }}n^{2}\left\{\underset{|i-j|=n}{\overset{n_{g}}{\underset{i=1}{\sum }}\overset{n_{g}}{\underset{j=1}{\sum }}p(i,j)}\right\}$$ (3)

$${function}_{entropy}=-\underset{i}{\sum }\underset{j}{\sum }p(i,j)log(p(i,j))$$ (4)

#### Bag of Words (BOW)

The Bag of Visual Words (BOVW or simply BOW) algorithm [18] is inspired in the Bag of Words technique used in text classification.

This technique was firstly developed to count the frequency of a set of words in a text in order to infer, for instance, the text main topic or subject. For example, in a text about plants it is expected to find more often the words flowers and leaves than in a description of a quantic theory. This way, BOW generates a histogram with the frequency of each word in each text that can be used to classify the text into a set of topics.

In order to extend the use of BOW to images, the concept of a visual word or a vocabulary of visual features have been introduced. A visual feature can be defined in many different ways, but the one used in this research is based on the Speeded up Robust Features (SURF) technique [19], that detects and describes interest points in an image based on the magnitude and direction of the gradients in and around each pixel of the image, discarding color information and using only the intensity (gray scale value). Regions of the image with higher gradient magnitudes in different directions are more likely to be chosen as an interest point. These regions correspond, for instance, to corners, crosses and curves and can be found, using SURF, even when the object is rotated or scaled.

Using a training dataset of images, a clustering algorithm like k-means is used to group all the interest points detected in all the training images in k groups. Each group is supposed to represent a set of visually similar regions and is called a visual word and the set of all k groups is called a visual vocabulary. Using this visual vocabulary, each image can now be represented by a histogram that counts the occurrence of each visual work in that image. Differently from the features used in CST, that somehow summarize global information about the pollen, BOW uses local information found in specific regions of the image.

### Color, Shape, Texture and BOW (CST+BOW)

The third technique explored in this work is just a combination or grouping of all the features extracted using CST and BOW and is called CST + BOW.

### Supervised Learning Techniques

The experiments with supervised learning techniques, also called classifiers, have been conducted using the Weka Software 3.7.9 [13]. Four classifiers available with Weka have been used: two variations of support vector machines, Sequential Minimal Optimization (SMO) and C-Support Vector Classification (C-SVC), a decision tree based classifier (J48) and the k-nearest neighbors (KNN).

The decision tree based classifier (J48) builds a hierarchical data set through the divide and conquer method. At each of the internal nodes of the tree, beginning with the root node, one feature is used to indicate a path that will lead to the most probable class of the example that is being analyzed. This path is followed until a leaf is reached and a decision about the most probable class for the example is taken [20].

The SVM is a type of linear classifier. This classifier defines a hyperplane that maintains a maximum margin which separates the categories or classes found from a data set of training examples. With the maximum margin defined and given a new example, the classification is made regarding the location of the new data into the maximum margin. SMO is, basically, a technique to speed-up the calculation of the quadratic programming (QP) problem that is central in the training or learning phase. It is a sequential method that solves analytically a series of smaller QP sub-problems derived from the original one [21, 22]. C-SVC is a realization of a soft-margin classifier where the constraints of the QP problem are relaxed to admit that some examples of a class overpass the hyperplane separating the classes. This is achieved by the introduction of slack variables and a constant C multiplying the summation of these variables in the QP objective function. Roughly speaking, the C constant, which is defined experimentally, controls how many examples and how deep into the other side of the hyperplane these examples can go [23].

The KNN is a type of lazy learning classifier where all the training images are simply memorized during the learning step. When a new test image is presented to the classifier, it is compared to each training image using a similarity measure and a rank is generated. The similarity measure used in our work is the Euclidean distance, which is a common choice in computer vision problems and is fast to calculate. The classes (e.g.: pollen types) of the K best ranked images are used to classify the new image by choosing for this new image the most frequent class among these K images.

To measure the performance of each technique in pollen identification, the following metrics were used: Correct Classification Rate (CCR), F-Measure and AUC. CCR is the percentage of pollen grains whose types are correctly classified. The F-Measure is a harmonic average among the true positives, false positives and false negatives, which measures the ability of the system to classify data [24]. The metric AUC uses the area under the ROC curve, and a larger area under the curve indicates a better performance [25]. Using the CCR metric, the data were expressed in a confusion matrix, which shows the correct pollen type classifications and the wrong classification cases obtained after the classification of the images. The confusion matrix was colored by adapting the thermal method, as reported previously [26].

A statistical analysis of the 12 combinations of techniques (3 feature extractors and 4 supervised learners) was performed using ANOVA and the R software [27]. Whenever significant differences were found (p<0.05), the Tukey’s post-test was used to analyze the techniques in a pairwise manner.

### Parameter Tuning

Some of the parameters of the feature extraction and supervised learning techniques have been tuned before testing. In special, for the BOW and the KNN techniques, the size of the dictionary and the value of K have been determined beforehand and in this section the methods used to find the values for these parameters are presented. No parameter tuning procedure was implemented for the other methods utilized in the experiments. In the case of J48, C-SVC and SMO, the default values available in Weka [13] were used and all the parameters related to the CST techniques were initialized with the values reported in [15].

Different vocabulary sizes were tested by exponentially growing the values from 2 to 4392 and evaluating the resulting performance, which has been reached with a 1536-sized vocabulary and at a 57% CCR. A finer test was then performed using arithmetic growth around the best vocabulary sizes defined before and a better performance of 60% CCR has been reached with a dictionary of 768 visual words. This value was used in the main experiment. Tests with the K values for the KNN classifier were conducted over a variation range of 1 to 13. The best performance was obtained with a K value of 1. Therefore, for the KNN classifier, the configuration that we adopted for the analysis of the techniques included a value of K equal to 1.

### Human Vision Classification

**Fig 3:**
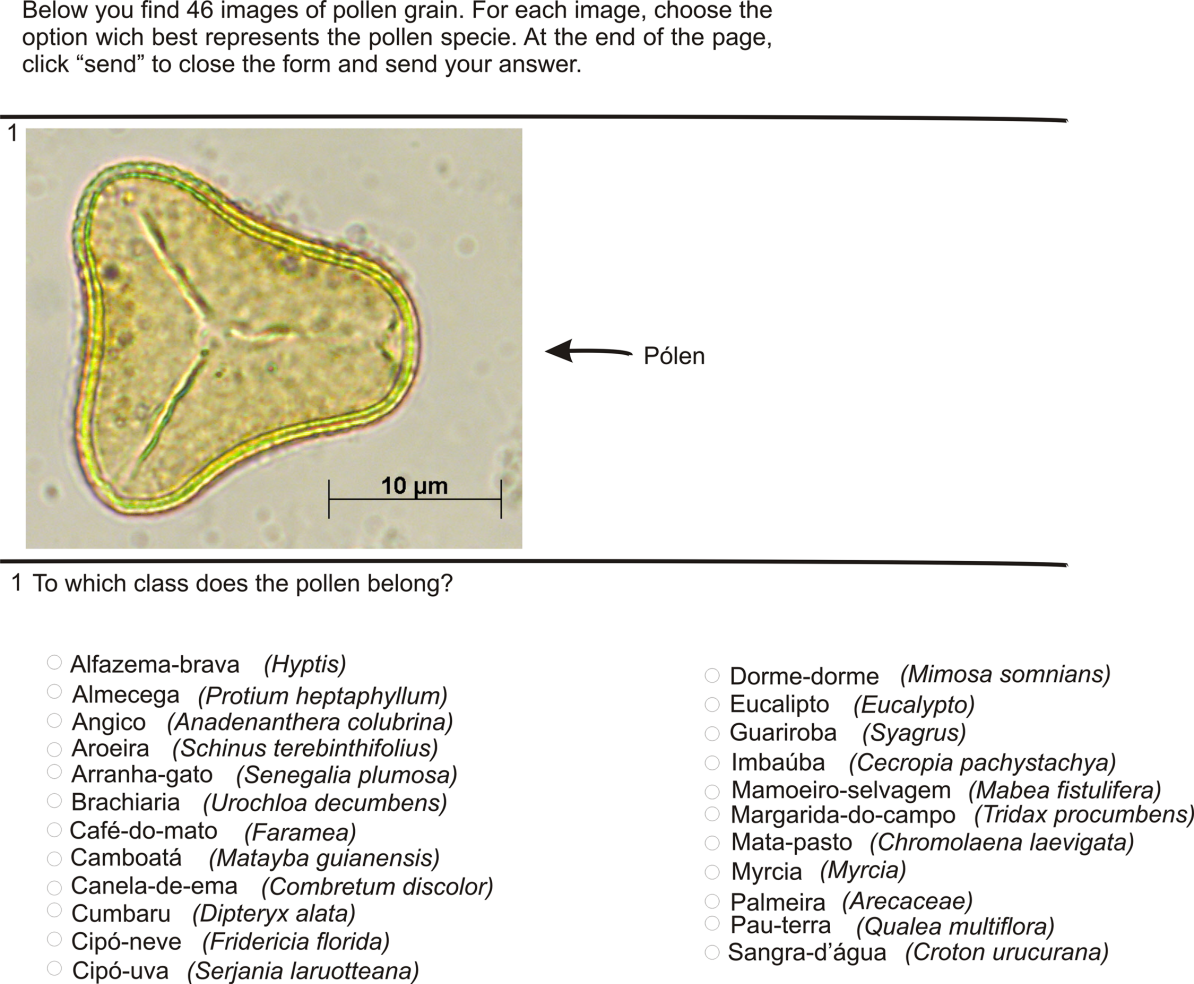
Part of the questionnaire containing a pollen image and the 23 pollen types options from which the beekeepers should select one.

A questionnaire (see S2 Text) with pollen images was applied to evaluate human performance in pollen classification. Twenty-three options of pollen types were available to be checked off in each displayed image. For the formulation of the questionnaire, images from the POLEN23E dataset were randomly selected for the test images. In addition, two different images of each pollen type were selected, what means that 46 images were selected to be identified (Fig 3). The common names of each pollen type were used as options to be marked to facilitate the assimilation of the images.

**Fig 4:**
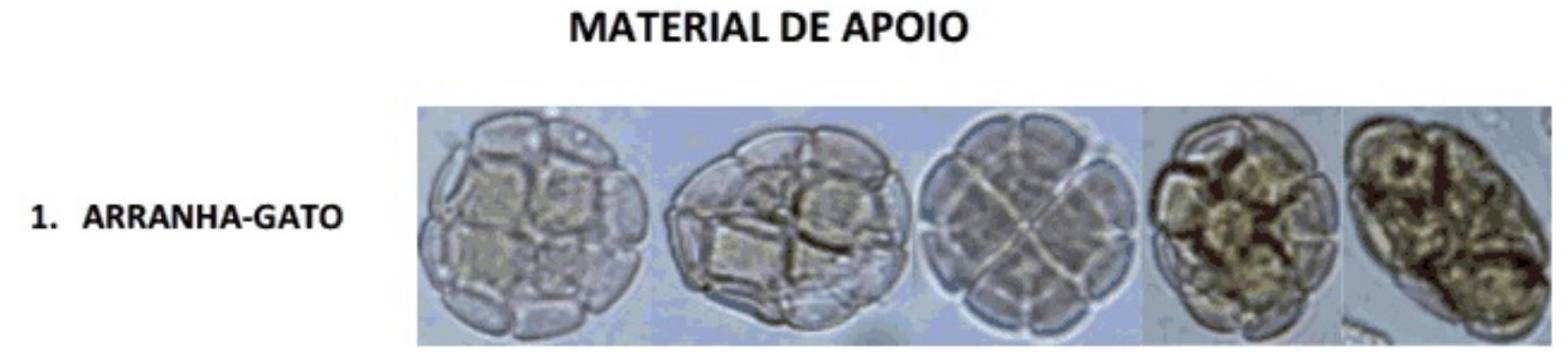
Five images of *Senegalia plumosa* pollen used in the support material. Beekeepers had access to this kind of reference material to answer the question during the application of the questionnaire. The common name of the *Senegalia plumosa* pollen type is “arranha-gato” in Portuguese.

From the POLEN23E dataset, five images of each pollen type were used for the training set, which comprised 115 images altogether. The volunteers were allowed to examine the support material (see S3 Text), while classifying the images, as shown in the questionnaire (Fig 4). In this material, the pollen common names were used because these are easier to memorize than their respective scientific names. The questionnaire also contained an open question where the users could write down the visual features that they analyzed in order to classify a pollen grain and the most difficult aspects of the classification process.

In this experiment, the questionnaire was applied to 34 volunteer beekeepers who did not have previous knowledge of the pollen identification process. They were advised to classify the 46 types of pollen in a virtual questionnaire. The answering time was recorded. At the end of the experiment, the answered questionnaire was saved and sent by e-mail to the researcher.

## Results

**Fig 5:**
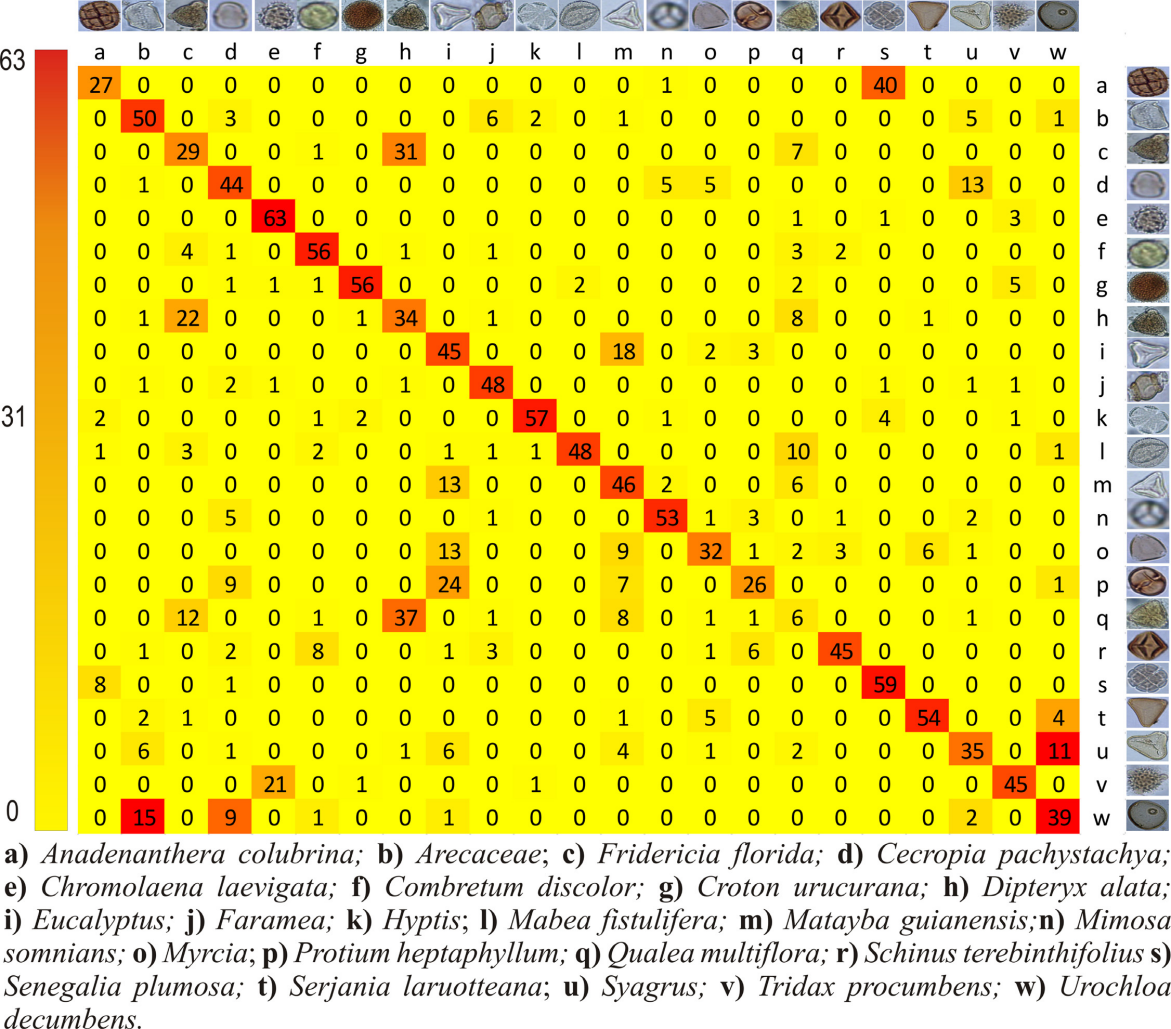
Confusion matrix summarizing the human performance on pollen classification. The rows represent the true pollen types while the columns indicate how the images have been classified by humans. All the correct answers are in the diagonal.

Fig 5 shows the number of hits and misses of pollen types that were classified by humans and indicates that 64% of the images were correctly classified (CCR of 64%). It is clear from the data that human performance varies a lot depending on the pollen types. The *Chromolaena laevigata* had the best accuracy of 92% while the pollen type *Q*. *multiflora* reached a correct classification rate of only 9%.

**Fig 6:**
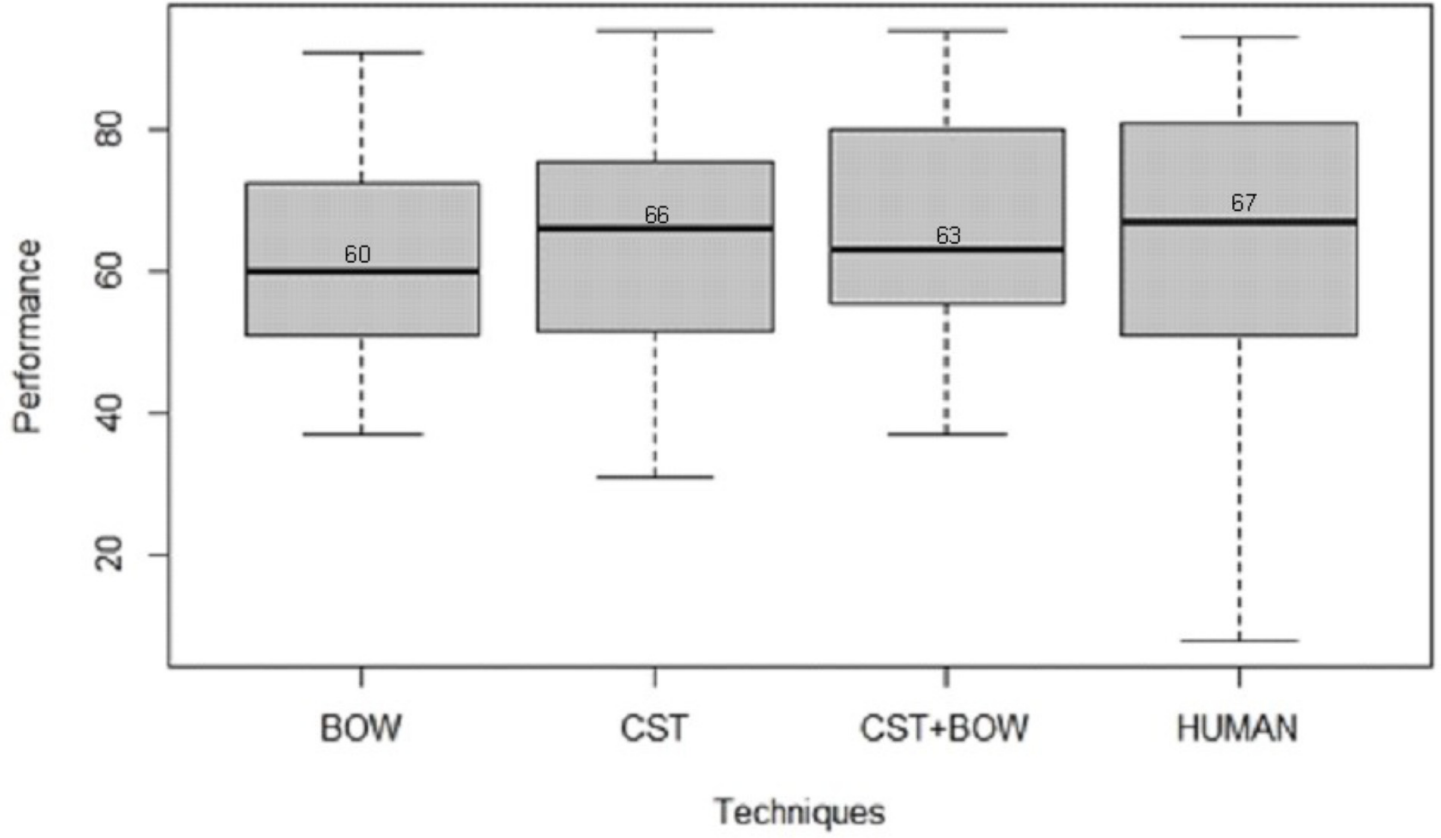
Boxplots for the CCR performance of the three feature extractors and the humans. Among the automatic techniques the highest median value was 66%, very near the median of the human performance, 67%.

**Fig 7:**
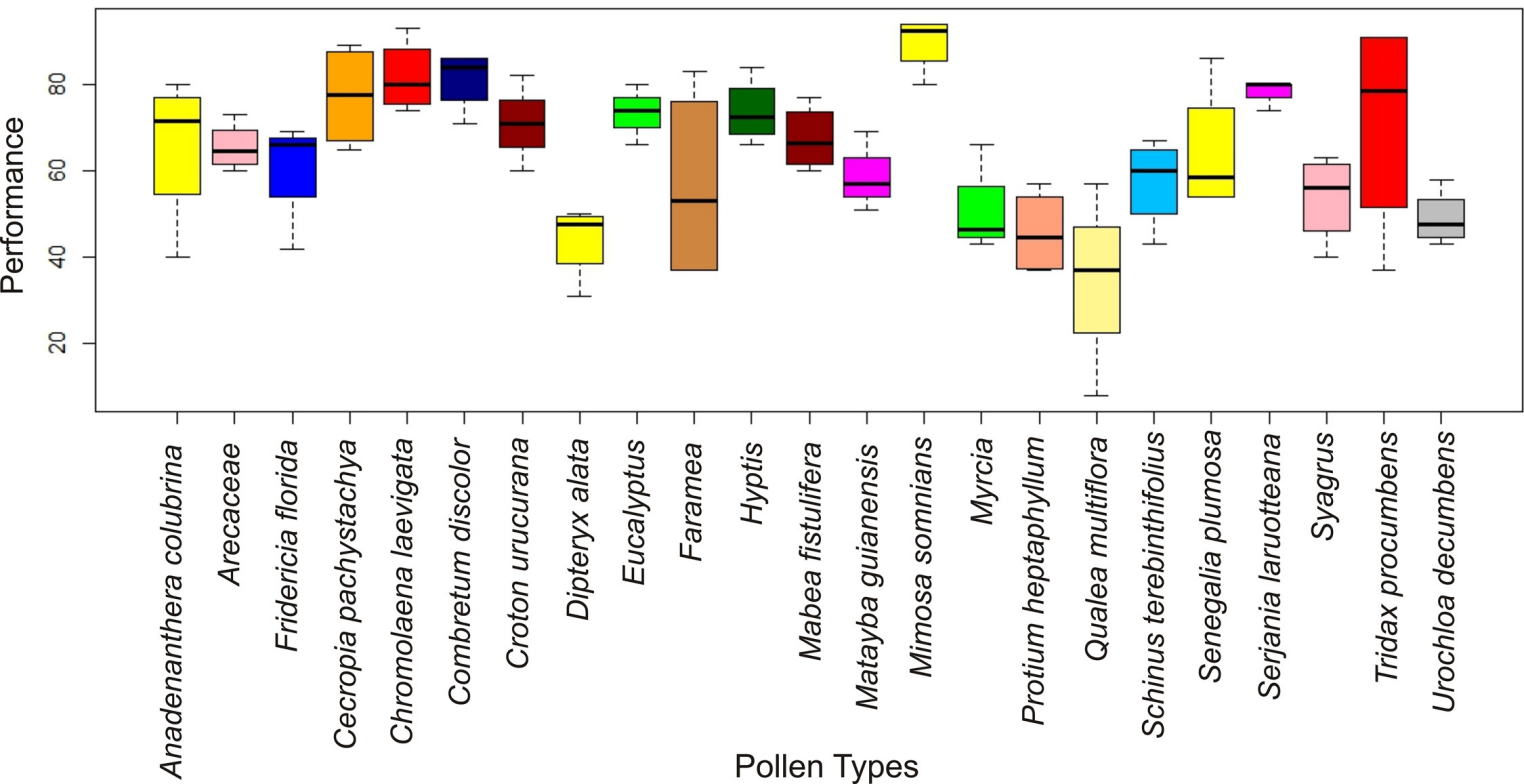
Boxplots for the CCR performance of the automatic techniques considering each pollen type. These boxplots show that automatic classification varies a lot among different pollen types.

As regarding the computer performance, no significant difference has been found (p-value = 0.1902) among the techniques explored. Fig 6 shows the boxplot diagram for the feature extraction techniques used in this research and the human performance. The p-value found, now considering human performance, was 0.576, which indicates no significant difference among computers and human performance. The higher p-value in this case is clearly linked with the high standard deviation on the tests with human. Fig 7 shows the CCRs for the computer performance separated by pollen types. In this case, ANOVA yielded a p-value of 0.000000125, indicating a clear dependence of the performance on the pollen types.

The analysis of 23 genera and species of 15 different families showed that a greater accuracy was not found for a specific family. The pollen features are very distinct among different genera, and there is no exclusive family feature set [1]. *Tridax procumbens* presented the highest variation in its classification, ranging from 37% to 91%. *Mimosa somnians* had a small accuracy variation, ranging from 80% to 94%, but it was the best classified pollen. The smallest accuracy variation, which ranged from 74% to 80%, was obtained for *Serjania laruotteana*.

Table 1 shows the results of the computational technique performance achieved by each combinations of feature extractor and classifier. The best performances for each metric are shown in bold. Uppercase letters indicate no significant difference among the feature extractors (rows) and lowercase letters represent no significant difference among the classifiers (column).

**Table 1:** Performance of each combination of techniques using three different metrics: CCR, F-Measure and AUC. For each combination and metric, the mean and standard deviation values are shown. The best performances for each metric are shown in bold. The same capital letters in the superscripts indicate no statistical difference between feature extractors (rows) as the same lower case letters indicate no significant difference between supervised learning techniques (columns).

		Supervised Learning			
Metrics	Feature Extraction	SMO	C-SVC	J.48	KNN
**CCR**	CST	48 ± 2.21^Bc^	63 ± 3.89^Aa^	54 ± 3.27^Ab^	60 ± 2.15^Aa^
	BOW	60 ± 2.58^Aa^	61 ± 2.59^Aa^	28 ± 3.27^Cb^	30 ± 2.02^Bb^
	CST + BOW	63 ± 2.26^Aa^	**64 ± 2.13**^**Aa**^	47 ± 1.92^Bb^	31 ± 2.42^Bc^
**F-Measure**	CST	46 ± 0.03^Cd^	63 ± 0.04^Ba^	54 ± 0.03^Ac^	60 ± 0.02^Ab^
	BOW	60 ± 0.03^Bb^	61 ± 0.03^Ca^	28 ± 0.03^Cd^	29 ± 0.02^Cc^
	CST + BOW	**64 ± 0.02**^**Aa**^	**64 ± 0.02**^**Aa**^	47 ± 0.02^Bb^	30 ± 0.02^Bc^
**AUC**	CST	95 ± 0.02^Ca^	83 ± 0.08^Bb^	76 ± 0.09^Ad^	79 ± 0.09^Ac^
	BOW	96 ± 0.04^Ba^	86 ± 0.09^Ab^	63 ± 0.09^Cc^	56 ± 0.02^Cd^
	CST + BOW	**97 ± 0.04**^**Aa**^	87 ± 0.08^Ab^	73 ± 0.05^Bc^	57 ± 0.03^Bd^

The highest CCR of 64% was obtained with CST+BOW and the C-SVC classifier, which achieved the highest CCR and F-Measure for every feature extractor combination. The performance using AUC was better for all of the feature extractors with the SMO classifier, all above 95%. It is well known in the computer vision field that the AUC is an over optimistic metric that almost always delivers higher values than F-Measure and CCR. It is clear from the results, and in accordance with the literature, that the support vector machine based classifiers (SMO and C-SVC) outperform KNN and J48 under all the metrics used.

In order to further investigate the performance of the techniques with the highest scores three confusion matrices have been produced, for each feature extractor combined with the C-SVC classifier. The main matrix diagonal represents the number of correctly classified images, and the off diagonal values represent errors in the classification.

**Fig 8:**
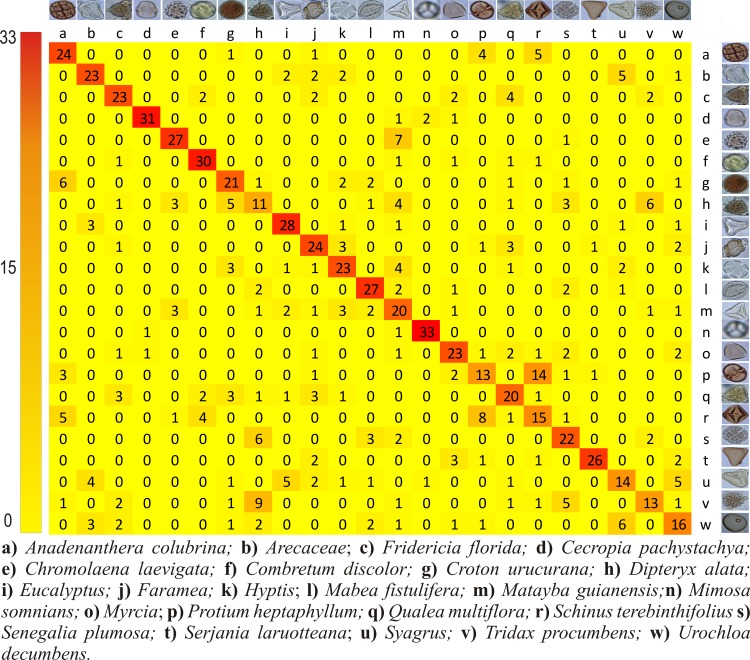
Confusion matrix for the best combinations of feature extractor (CST) and classifier (C-SVC). The rows represent the true pollen types while the columns indicate how the images have been classified by the computer. All the correct answers are in the diagonal.

Fig 8 shows that, using CST, the pollen type *M*. *somnians* was mistakenly classified only twice, with 33 correct classifications. The *D*. *alata* pollen presented the worst classification with only 11 images being correctly classified. The pollen images of *C*. *pachystachya* had 31 hits (85.57%) with this technique, a percentage that is very close (89%) to that obtained by [4] in a study on *Urticaceae* pollen grains.

**Fig 9:**
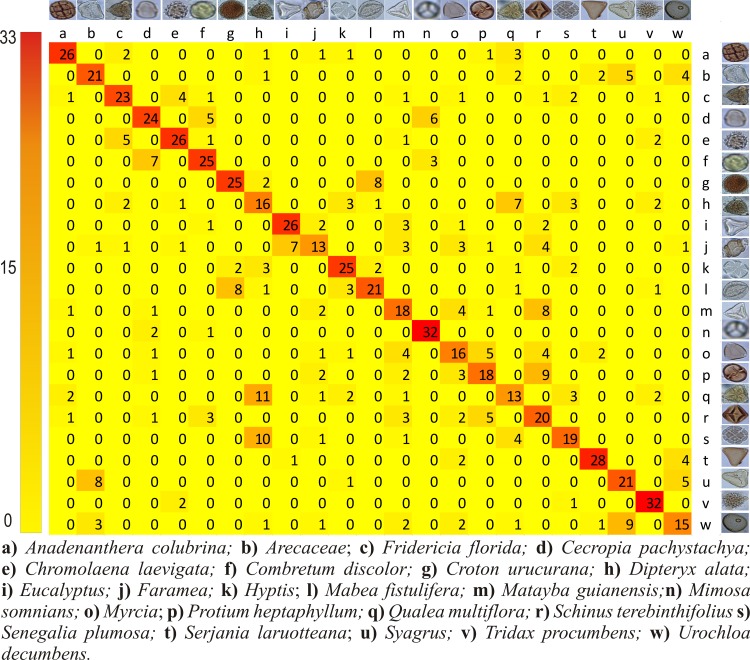
Confusion matrix for the combination of BOW and C-SVC. The rows represent the true pollen types while the columns indicate how the images have been classified by the computer. All the correct answers are in the diagonal.

**Fig 10:**
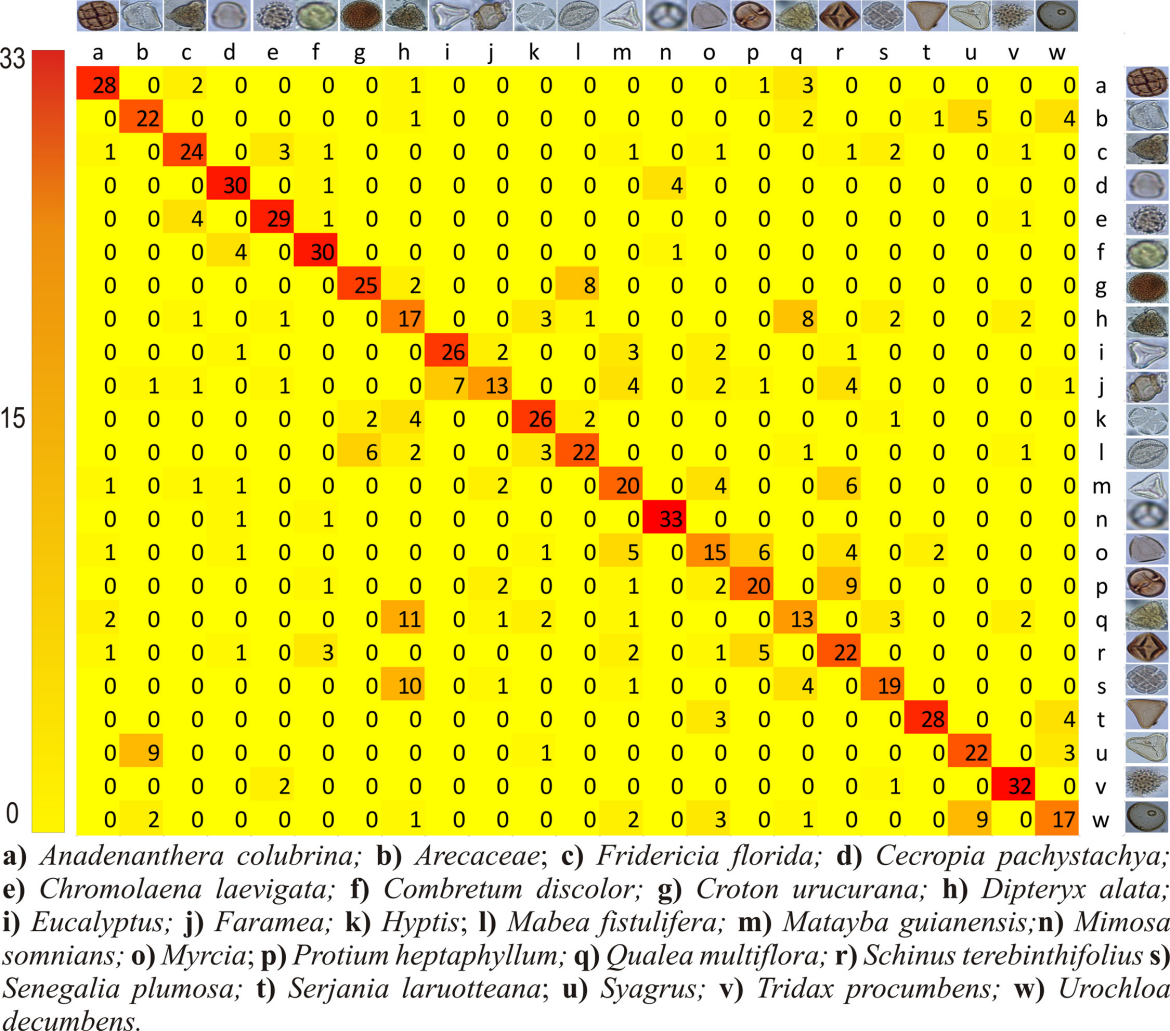
Confusion matrix for the combination the CST+BOW feature extractor and C-SVC. The rows represent the true pollen types while the columns indicate how the images have been classified by the computer. All the correct answers are in the diagonal.

With the BOW technique, the *M*. *somnians* and *T*. *procumbens* pollen grains were correctly classified 32 times (91%), whereas the *Faramea* and *Q*. *multiflora* pollen grains were the worst classified pollen grains, with only 37% of their images being correctly classified (Fig 9). Using the CST + BOW technique (Fig 10), *M*. *somnians* presented the highest percentage of correct classifications, which equaled 94% (33 hits), whereas *Faramea* and *Q*. *multiflora* presented the lowest percentages of correct classifications, which was only 37% (13 hits).

## Discussion

Through the questionnaire applied to the beekeepers, it was possible to know which features the beekeepers used for pollen classification. The beekeepers reported that the main feature observed was the shape, in addition to the color, size and texture features. These characteristics were also used by the computational techniques. Although the human classification was closely aligned with the automatic techniques, the beekeepers spent almost two hours, on average, finishing the questionnaire. The automated techniques required less than 10 seconds, on average, to classify the pollen grains.

Comparing computers and humans performance regarding the time to complete a task is very difficult and the results must be always used with caution. In our experiments, the training and testing examples have been randomly chosen from the same POLEN23E dataset, for both computer and humans, and we also have used roughly the same amount of images in the test set for both. In the case of computers, a three fold cross-validation has been used and so three different sets of training and test images were used, whereas in the case of humans, only one random training and test image set were used. This happened because the time we had with the group of humans was limited. As not all beekeepers are trained to use computers, part of the time they took to fill the online questionnaire can be associated with tasks that are not directly related to the classification process but, for instance, to move the computer mouse to click a button. Before the experiment, they have been instructed on how to use the questionnaire and did some training to reduce this problem of the lack of computer skills influencing the time to complete the task.

**Fig 11:**
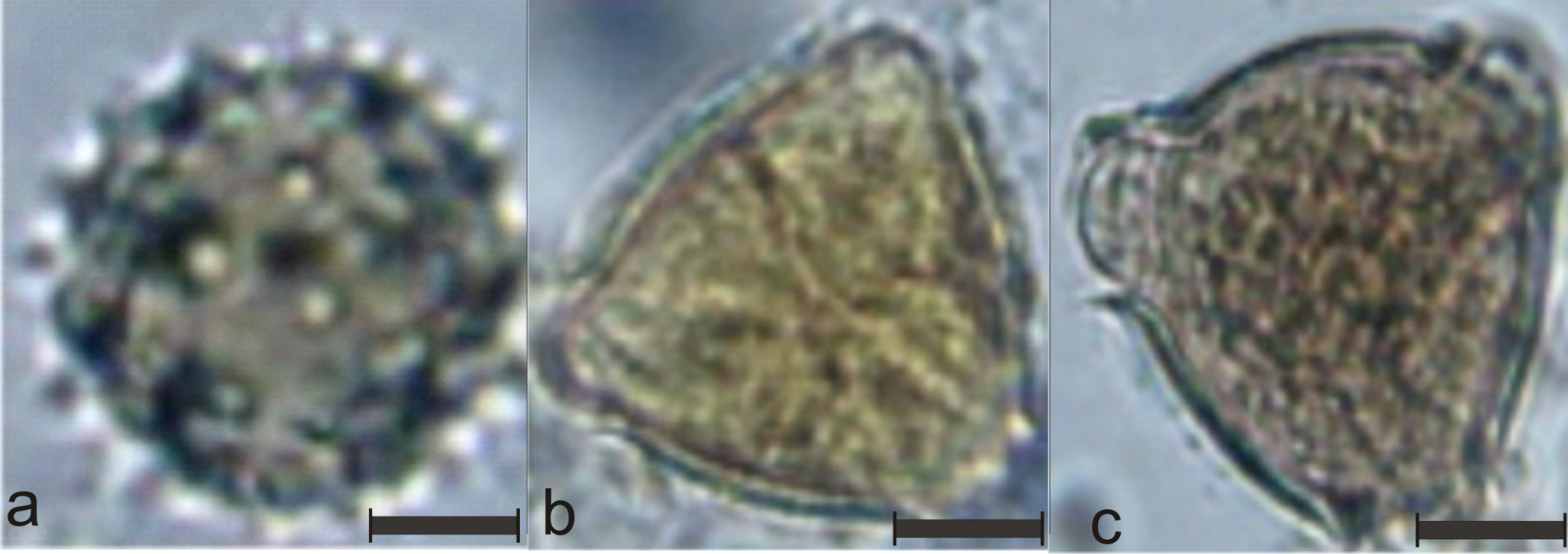
Examples of the most easy and most difficult pollen types for human classification. a) One example of the *Chromolaena laevigata* pollen that received the highest CCR score for human performance; b) One example of the *Qualea multiflora* pollen, the hardest pollen during human classification c) Example of the *Dipteryx alata* pollen that has been mostly confused with *Qualea multiflora*.

The pollen type that was best classified by the beekeepers, as shown in Fig 5, was *C*. *laevigata* (Fig 11A). This type has a rounded shape with spines on its surface, which helped the beekeepers to recognize it. The beekeepers mentioned difficulties during pollen classification with regard to seeing similar shapes, having little perception of the pollen size, seeing unclear images and having many views of the same pollen.

As shown in Fig 5, *Q*. *multiflora* presented the worst CCR (9%). The reason for this low performance could be that the pollen is very similar to *D*. *alata* pollen from all points of view, as seen in Fig 11B and 11C. Additionally, 54% of the images of *Q*. *multiflora* were misidentified as *D*. *alata*.

Both CST (Fig 8), BOW (Fig 9) and CST + BOW (Fig 10) techniques faced problems to identify the same pollen types that were most often mistakenly classified by the beekeepers, the *Q*. *multiflora* and the *D*. *alata*. More images from different angles are needed, and more details should be captured to allow distinction between these pollen grains.

In the pollen classification by computational techniques, all of the features were efficient for extracting information from the *M*. *somnians* images. This pollen type presented the best image classification performance with all of the automatic techniques. It is easy to perceive that there are many interesting high-contrast corners in the images of *M*. *somnians* that could be easily detected by the BOW feature extractor. This pollen type has also some very distinctive colors and shapes that facilitate its identification regarding the texture feature, there is a clear perception that the cracks in pollen grain are helpful for the extraction of this feature.

As shown in Table 1, BOW achieved a performance of 96% when measured via the AUC metric, with 0.4 of standard deviation. This value was close to the performance that was obtained in a previous study [9] that sought to classify pollen types of *Betula* using apertures. These researchers used the BOW technique with a dictionary with 184 descriptors, 92 images of pollen apertures and 92 images without aperture. The performance was also analyzed with SVM, and the result was evaluated with the AUC metric. The best performance obtained was 95.8%. In the present research, the BOW performance was similar to that obtained by those researchers, which proves the efficiency of this technique for the extraction of features from images. In our study, 23 pollen types from different genera were used, and the performance measured with the AUC metric was similar to that obtained by other researchers, who assessed only one genus.

In researches aiming to automate the classification of pollen grains, the performances reported by [2] with three species achieved 90% with the technique of neural network. [4] have used four pollen types and reached 94% with the single classification class technique. [6] had 70% with nine pollen types with the BOW technique, and [8] obtained 90% in classification of 17 pollen types with neural network. We obtained a performance of 64% with the CST+BOW technique. In spite of the general low performance compared to studies that utilized less pollen types [2, 4, 6, 8], seven pollen types that we classified with the CST+BOW technique had performance ≥80%, *A*. *colubrina* and *S*. *laruotteana* (80%), *C*. *laevigata* (82%), *C*. *pachystachya* (86%), *T*. *procumbens* (91%), *C*. *discolor* and *M*. *somnians* (94%). In the work of [28], which used the technique of pollen grain recognition in 3D on 30 pollen types, those authors achieved a performance of 77% in pollen classification. Thus, either in our research as in [28], we can perceive in relation to the works of [2, 4, 6, 7, 8] that when the number of pollen types utilized in automatic classification increases, the performance tends to decrease.

Previous attempts to automate pollen identification have been reported, but the novelty of our research is that it includes the largest number of tropical pollen types used in a study so far. Seventeen tropical pollen types from Costa Rica was the highest quantity found for automatic classification of pollen images obtained under LM [8]. Moreover, many of the features associated with color, shape and texture were not used in our research study, and even with the limited number of chosen features, we obtained a pollen classification accuracy greater than 64%. Hence, the results with the techniques presented in this study are quite promising.

## Conclusions

A new public dataset of pollen images, together with the baseline performance of humans to classify images from this dataset, has been made available. Several computer vision and machine learning techniques have been explored in order to automate the process of pollen classification using these new dataset. These techniques can rapidly classify pollen images into their respective types, which indicates the feasibility of using computer for this identification task, especially considering that this research included as many as 23 different types of pollen. It is still necessary to improve the image sharpness of the pollen structures to achieve better classification by the program.

The best technique that should be used to automate pollen classification is CST+BOW with the C-SVC classifier. A software for automatic pollen recognition can contribute to the knowledge of the local flora, the botanical origin of bee products and other important fields, such as forensic science and allergology.

## Future Work

One method for sharpening images is to stack multiple images of the same pollen with changes in only the focus. Thus, using a program such as ImageJ, it is possible to stack all of the captured images of the grain and create a single image that is acquired with the details obtained from the stacked images.

**Fig 12:**
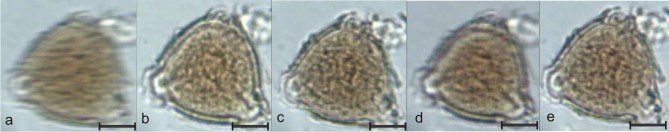
*Dipteryx alata* image stacking. a-d) Images from the same pollen grain. e) Sharp image obtained by stacking the images shown in a-d. To solve the problem of blurred images, the stacking of different focuses of the pollen image can be a promising approach.

For future research, we suggest using this stacking method for image capturing. This procedure does not allow any loss of details of the pollen grains, and even blurred images become sharp by grouping the images. Fig 12 shows this process in the pollen of *D*. *alata*, from which four images were captured only by changing the focus. In Fig 12E, it is possible to see the result of the stacking process of the images of *D*. *alata* and the details of this grain.

## Supporting Information

S1 Dataset POLEN23E (http://dx.doi.org/10.6084/m9.figshare.1525086). (PDF)

S1 Text Declaration of the President of the Beekeeping and Meliponiculture Federation–Mato Grosso do Sul (FEAMS) authorizing the questionnaire application for beekeepers (http://dx.doi.org/10.6084/m9.figshare.1529340). (PDF)

S2 Text Questionnaire with pollen images (http://dx.doi.org/10.6084/m9.figshare.1529342). (PDF)

S3 Text Support material with pollen images (http://dx.doi.org/10.6084/m9.figshare.1529341). (PDF)
