# Supplementary material for: Feature Extraction and Machine Learning for the Classification of Brazilian Savannah Pollen Grains
Source: PLoS One. 2016 Jun 8;11(6):e0157044. doi: 10.1371/journal.pone.0157044 (PMC4898734; doi:10.1371/journal.pone.0157044)
Supplement: S1 Dataset — (PDF) [file pone.0157044.s001.pdf]

My data search figshare (titles, tags, authors, etc.) Browse Upload A. Barbosa Gonçal

POLEN23E

Edit article

anadenanthera_17.jpg	preview	download
arecaceae_02.jpg	preview	download
arrabidaea_03.jpg	preview	download
cecropia_02.jpg	preview	download
chromolaena_01.jpg	preview	download
combretum_03.jpg	preview	download
croton_02.jpg	preview	download

Download all

20 views 0 shares

cites coming soon

Published on **28 Aug 2015 - 02:12 (GMT)**
Filesize in total is **32.85 MB**

Categories

Palynology
Botany
Computer Software
Applied Computer Science

Authors

Ariadne Barbosa Gonçalves

Tags

savannah pollen
pollengrain palynology
Extreme Learning Machine
computer vision

License (what's this?)

CC-BY

Export

Export to RefWorks
Export to BibTeX
Export to Ref. manager
Export to Mendeley
Export to Endnote
Export to DataCite
Export to NLM
Export to DC

Share this: g+1 0 Embed*

Cite this: Barbosa Gonçalves, Ariadne (2015): POLEN23E. fig**share**.
http://dx.doi.org/10.6084/m9.figshare.1525086
Retrieved 16:09, Sep 01, 2015 (GMT)

*The embed functionality can only be used for non commercial purposes... more

Description

Image dataset for training and testing computer vision software for pollen identification.

Anadenanthera colubrina - http://dx.doi.org/10.6084/m9.figshare.1524247

Arecaceae - http://dx.doi.org/10.6084/m9.figshare.1524283

Arrabidaea florida - http://dx.doi.org/10.6084/m9.figshare.1524320

Cecropia pachystachya - http://dx.doi.org/10.6084/m9.figshare.1524356

Chromolaena laevigata - http://dx.doi.org/10.6084/m9.figshare.1524392

Combretum discolor - http://dx.doi.org/10.6084/m9.figshare.1524428

Croton urucurana - http://dx.doi.org/10.6084/m9.figshare.1524464

Dipteryx alata - http://dx.doi.org/10.6084/m9.figshare.1524501

Eucalyptus - http://dx.doi.org/10.6084/m9.figshare.1524537

Faramea - http://dx.doi.org/10.6084/m9.figshare.1524573

Hyptis - http://dx.doi.org/10.6084/m9.figshare.1524609

Mabea fistulifera - http://dx.doi.org/10.6084/m9.figshare.1524645

Matayba guianensis - http://dx.doi.org/10.6084/m9.figshare.1524681

Mimosa somnians - http://dx.doi.org/10.6084/m9.figshare.1524753

Myrcia - http://dx.doi.org/10.6084/m9.figshare.1524789

Protium heptaphyllum - http://dx.doi.org/10.6084/m9.figshare.1524826

Qualea multiflora - http://dx.doi.org/10.6084/m9.figshare.1524862

Schinus terebinthifolius - http://dx.doi.org/10.6084/m9.figshare.1524898

Senegalia plumosa - http://dx.doi.org/10.6084/m9.figshare.1524935

Serjania laruotteana - http://dx.doi.org/10.6084/m9.figshare.1524972

Syagrus - http://dx.doi.org/10.6084/m9.figshare.1525008

Tridax procumbens - http://dx.doi.org/10.6084/m9.figshare.1525046

Urochloa decumbens - http://dx.doi.org/10.6084/m9.figshare.1525085

Comments (0)

Post a new comment

Post comment

About | Features | Upgrade | Tools | Advisors | Blog | Contact | FAQs | Privacy Policy | Terms & Conditions

figshare. credit for all your research.
