# Supplementary material for: Feature Extraction and Machine Learning for the Classification of Brazilian Savannah Pollen Grains
Source: PLoS One. 2016 Jun 8;11(6):e0157044. doi: 10.1371/journal.pone.0157044 (PMC4898734; doi:10.1371/journal.pone.0157044)
Supplement: S1 Text — (PDF) [file pone.0157044.s002.pdf]

# DECLARATION

I, Gustavo Nadeu Bijos, CPF 639.952.711-20, the President of the Beekeeping and Meliponiculture Federation of Mato Grosso do Sul (FEAMS) CNPJ 12.365.889/0001-90, I DECLARE for the purposes of provide the ethics statement consent require for the research entitled Feature Extraction and Machine Learning for Brazilian Savanna Pollen Classification, as the Master's Thesis theme of the first author, the Biology Ariadne Barbosa Gonçalves and his co-authors, that the researche informations were obtained in interviews with beekeepers registered in this representative organization, of the state's producers, with full consent of all them. As President of the Beekeeping Federation I also certify that the interviews were scheduled by the interests of beekeepers, who consider the research subject to identify the botanic species whose pollen are present in honey samples provided with the purpose of disposal to the beekeepers an easy, fast, cheap and reliable method to value the honey in the domestic and international market. Finally I DECLARE that no information provided freely caused any embarrassment or personal information of interviewed beekeepers were used.

Campo Grande, 09, January, 2012.

Gustavo Nadeu Bijos – CRMV/MS 2100

President of the Beekeeping and Meliponiculture Federation - Mato Grosso do Sul (FEAMS)
