# Supplementary material for: Feature Extraction and Machine Learning for the Classification of Brazilian Savannah Pollen Grains
Source: PLoS One. 2016 Jun 8;11(6):e0157044. doi: 10.1371/journal.pone.0157044 (PMC4898734; doi:10.1371/journal.pone.0157044)
Supplement: S2 Text — (PDF) [file pone.0157044.s003.pdf]

Editar este formulário

Pólen

Temos a seguir 46 imagens de grãos de pólen. Para cada imagem, escolha (clicando) a opção que melhor representa a espécie do grão de pólen. Ao final da página, clique no botão enviar para fechar o formulário e enviar as suas respostas.

1

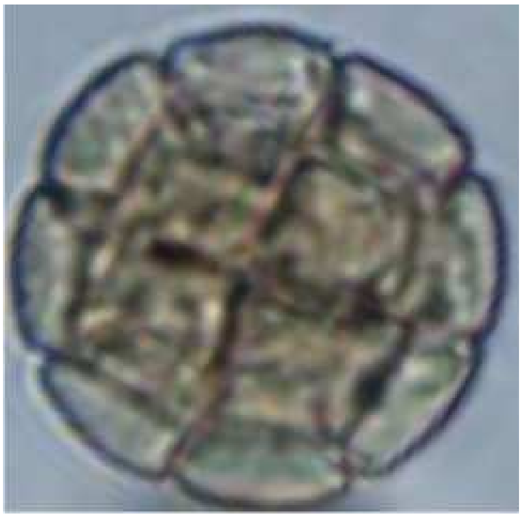

1

A que classe este pólen pertence?

- Alfazema-brava
- Almecega
- Angico
- Aroeira
- Arranha-gato
- Brachiaria
- Café-do-mato
- Camboatá
- Canela-de-ema
- Carvão-vermelho
- Cipó-neve
- Cipó-uva
- Dorme-dorme
- Eucalipto
- Guariroba
- Imbaúba
- Mamoeiro-selvagem

- Margarida-do-campo
- Mata-pasto
- Myrcia
- Palmeira
- Pau-terra
- Sangra d' água

2

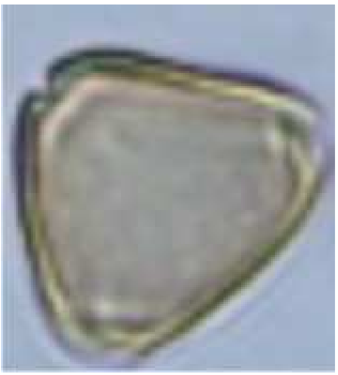

2

A que classe este pólen pertence?

- Alfazema-brava
- Almecega
- Angico
- Aroeira
- Arranha-gato
- Brachiaria
- Café-do-mato
- Camboatá
- Canela-de-ema
- Carvão-vermelho
- Cipó-neve
- Cipó-uva
- Dorme-dorme
- Eucalipto
- Guariroba
- Imbaúba
- Mamoeiro-selvagem
- Margarida-do-campo
- Mata-pasto
- Myrcia
- Palmeira
- Pau-terra
- Sangra d' água

3

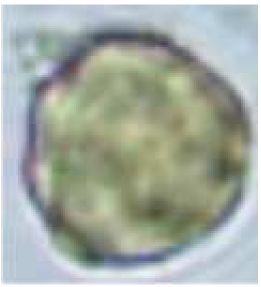

3

A que classe este pólen pertence?

- Alfazema-brava
- Almecega
- Angico
- Aroeira
- Arranha-gato
- Brachiaria
- Café-do-mato
- Camboatá
- Canela-de-ema
- Carvão-vermelho
- Cipó-neve
- Cipó-uva
- Dorme-dorme
- Eucalipto
- Guariroba
- Imbaúba
- Mamoeiro-selvagem
- Margarida-do-campo
- Mata-pasto
- Myrcia
- Palmeira
- Pau-terra
- Sangra d' água

4

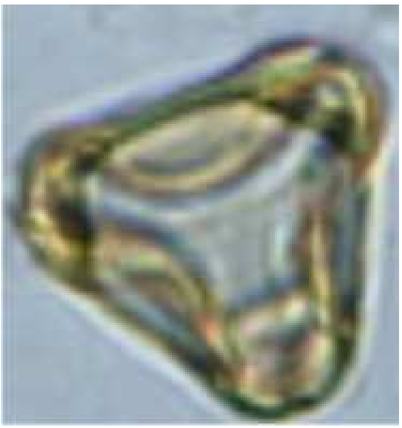

4

A que classe este pólen pertence?

- Alfazema-brava
- Almecega
- Angico
- Aroeira
- Arranha-gato
- Brachiaria
- Café-do-mato
- Camboatá
- Canela-de-ema
- Carvão-vermelho
- Cipó-neve
- Cipó-uva
- Dorme-dorme
- Eucalipto
- Guariroba
- Imbaúba
- Mamoeiro-selvagem
- Margarida-do-campo
- Mata-pasto
- Myrcia
- Palmeira
- Pau-terra
- Sangra d' água

5

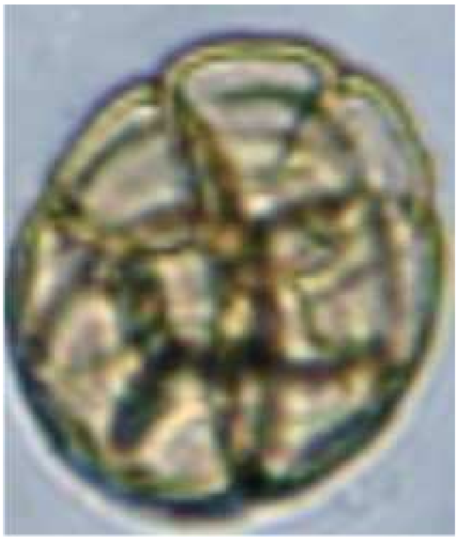

5

A que classe este pólen pertence?

- Alfazema-brava
- Almecega
- Angico
- Aroeira
- Arranha-gato
- Brachiaria
- Café-do-mato
- Camboatá
- Canela-de-ema
- Carvão-vermelho
- Cipó-neve
- Cipó-uva
- Dorme-dorme
- Eucalipto
- Guariroba
- Imbaúba
- Mamoeiro-selvagem
- Margarida-do-campo
- Mata-pasto
- Myrcia
- Palmeira
- Pau-terra
- Sangra d' água

6

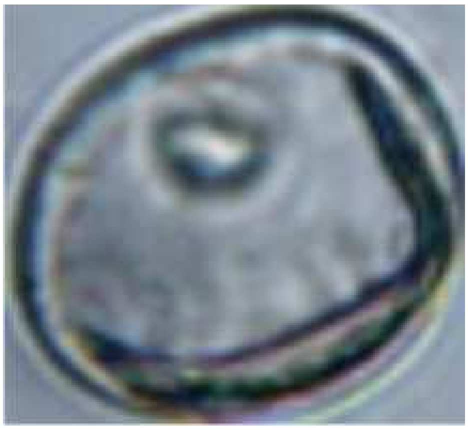

6

A que classe este pólen pertence?

- Alfazema-brava
- Almecega
- Angico
- Aroeira
- Arranha-gato
- Brachiaria
- Café-do-mato
- Camboatá
- Canela-de-ema
- Carvão-vermelho
- Cipó-neve
- Cipó-uva
- Dorme-dorme
- Eucalipto
- Guariroba
- Imbaúba
- Mamoeiro-selvagem
- Margarida-do-campo
- Mata-pasto
- Myrcia
- Palmeira
- Pau-terra
- Sangra d' água

7

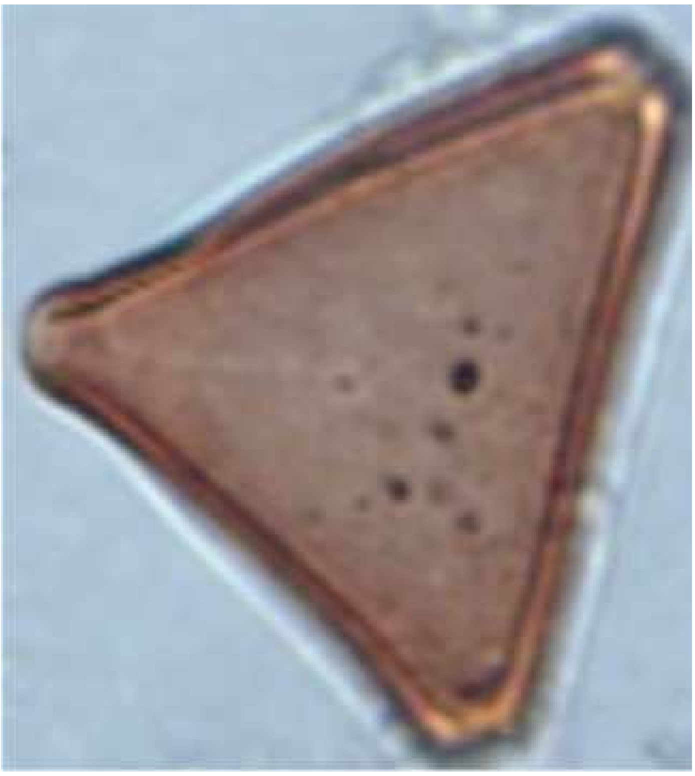

7

A que classe este pólen pertence?

- Alfazema-brava
- Almecega
- Angico
- Aroeira
- Arranha-gato
- Brachiaria
- Café-do-mato
- Camboatá
- Canela-de-ema
- Carvão-vermelho
- Cipó-neve
- Cipó-uva
- Dorme-dorme
- Eucalipto
- Guariroba
- Imbaúba
- Mamoeiro-selvagem
- Margarida-do-campo
- Mata-pasto
- Myrcia
- Palmeira
- Pau-terra
- Sangra d' água

8

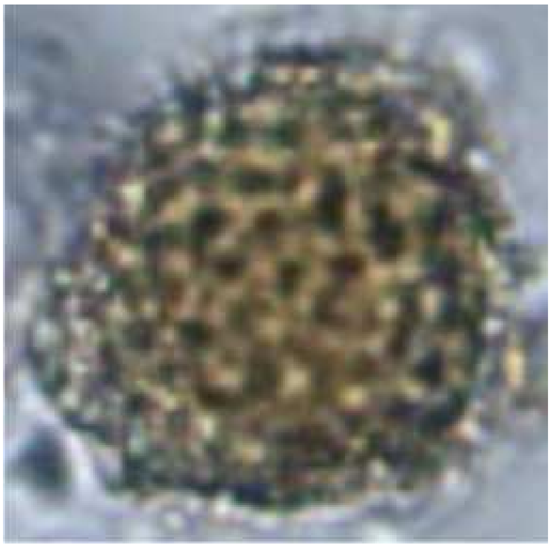

8

A que classe este pólen pertence?

- Alfazema-brava
- Almecega
- Angico
- Aroeira
- Arranha-gato
- Brachiaria
- Café-do-mato
- Camboatá
- Canela-de-ema
- Carvão-vermelho
- Cipó-neve
- Cipó-uva
- Dorme-dorme
- Eucalipto
- Guariroba
- Imbaúba
- Mamoeiro-selvagem
- Margarida-do-campo
- Mata-pasto
- Myrcia
- Palmeira
- Pau-terra
- Sangra d' água

9

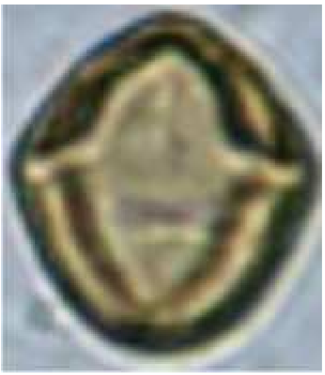

9

A que classe este pólen pertence?

- Alfazema-brava
- Almecega
- Angico
- Aroeira
- Arranha-gato
- Brachiaria
- Café-do-mato
- Camboatá
- Canela-de-ema
- Carvão-vermelho
- Cipó-neve
- Cipó-uva
- Dorme-dorme
- Eucalipto
- Guariroba
- Imbaúba
- Mamoeiro-selvagem
- Margarida-do-campo
- Mata-pasto
- Myrcia
- Palmeira
- Pau-terra
- Sangra d' água

10

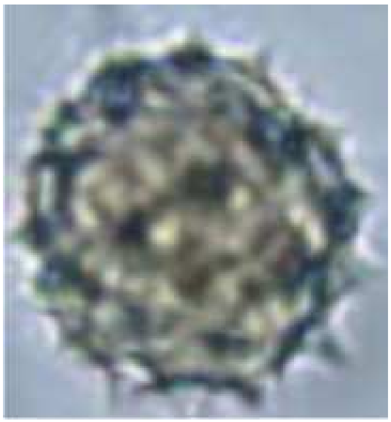

10

A que classe este pólen pertence?

- Alfazema-brava
- Almecega
- Angico
- Aroeira
- Arranha-gato
- Brachiaria
- Café-do-mato
- Camboatá
- Canela-de-ema
- Carvão-vermelho
- Cipó-neve
- Cipó-uva
- Dorme-dorme
- Eucalipto
- Guariroba
- Imbaúba
- Mamoeiro-selvagem
- Margarida-do-campo
- Mata-pasto
- Myrcia
- Palmeira
- Pau-terra
- Sangra d' água

11

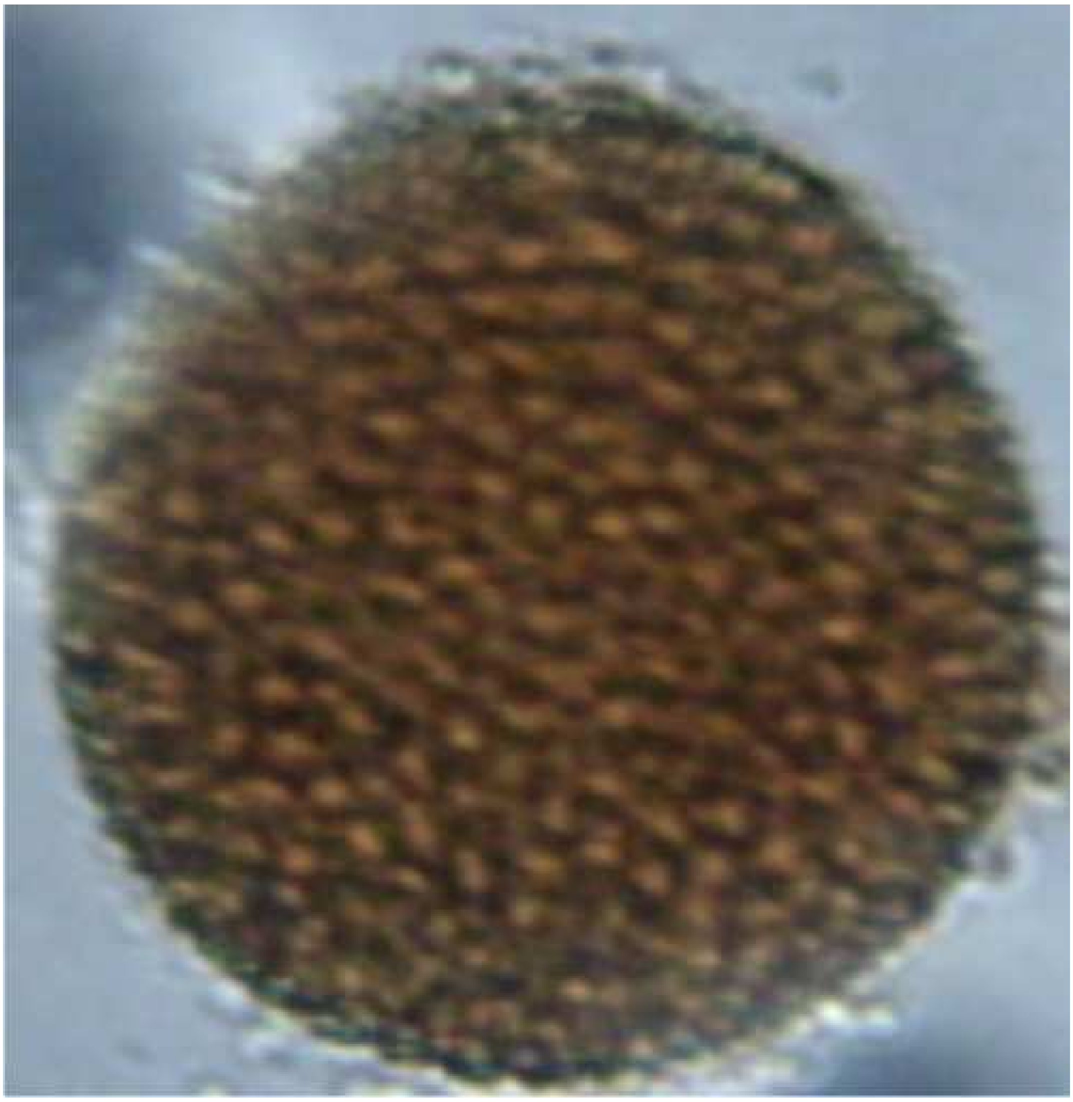

11

A que classe este pólen pertence?

- Alfazema-brava
- Almecega
- Angico
- Aroeira
- Arranha-gato
- Brachiaria
- Café-do-mato
- Camboatá
- Canela-de-ema
- Carvão-vermelho
- Cipó-neve
- Cipó-uva
- Dorme-dorme
- Eucalipto
- Guariroba
- Imbaúba
- Mamoeiro-selvagem

- Margarida-do-campo
- Mata-pasto
- Myrcia
- Palmeira
- Pau-terra
- Sangra d' água

12

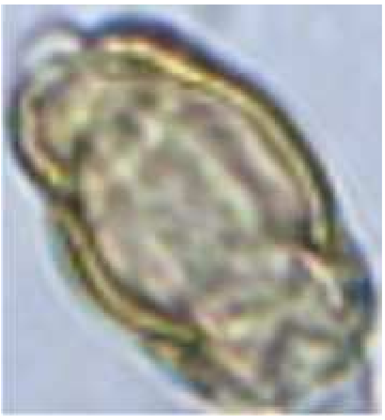

12

A que classe este pólen pertence?

- Alfazema-brava
- Almecega
- Angico
- Aroeira
- Arranha-gato
- Brachiaria
- Café-do-mato
- Camboatá
- Canela-de-ema
- Carvão-vermelho
- Cipó-neve
- Cipó-uva
- Dorme-dorme
- Eucalipto
- Guariroba
- Imbaúba
- Mamoeiro-selvagem
- Margarida-do-campo
- Mata-pasto
- Myrcia
- Pau-terra
- Palmeira

- Sangra d' água

13

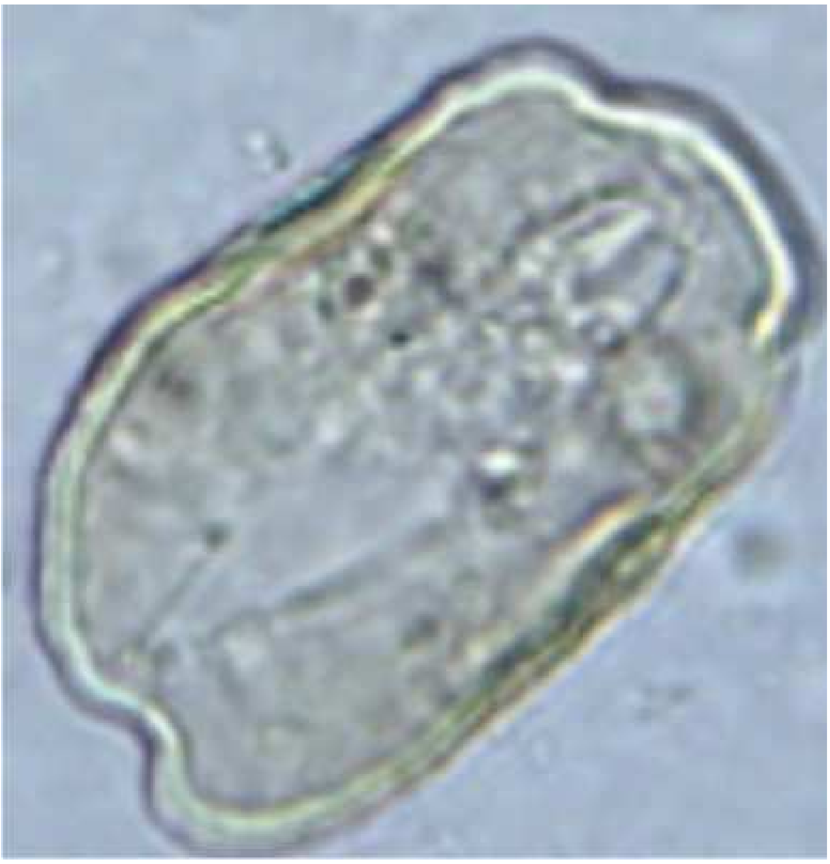

13

A que classe este pólen pertence?

- Alfazema-brava
- Almecega
- Angico
- Aroeira
- Arranha-gato
- Brachiaria
- Café-do-mato
- Camboatá
- Canela-de-ema
- Carvão-vermelho
- Cipó-neve
- Cipó-uva
- Dorme-dorme
- Eucalipto
- Guariroba
- Imbaúba
- Mamoeiro-selvagem
- Margarida-do-campo

- Mata-pasto
- Myrcia
- Palmeira
- Pau-terra
- Sangra d' água

14

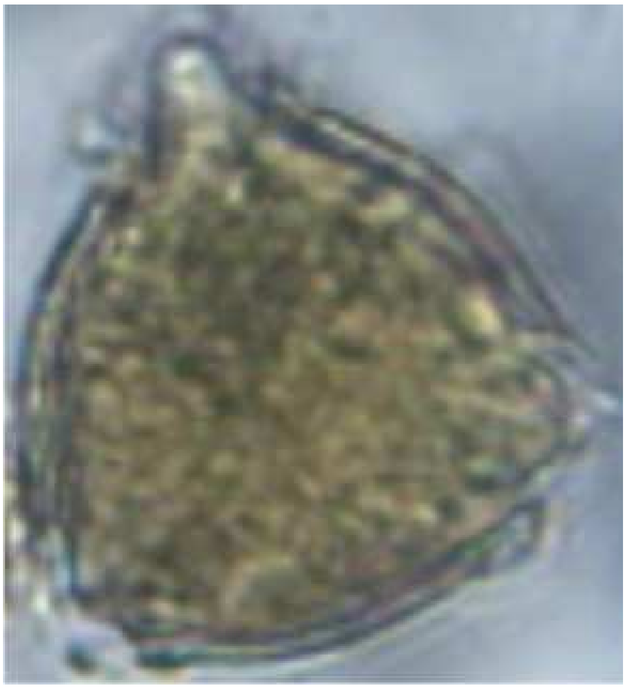

14

A que classe este pólen pertence?

- Alfazema-brava
- Almecega
- Angico
- Aroeira
- Arranha-gato
- Brachiaria
- Café-do-mato
- Camboatá
- Canela-de-ema
- Carvão-vermelho
- Cipó-neve
- Cipó-uva
- Dorme-dorme
- Eucalipto
- Guariroba
- Imbaúba
- Mamoeiro-selvagem
- Margarida-do-campo

- Mata-pasto
- Myrcia
- Palmeira
- Pau-terra
- Sangra d' água

15

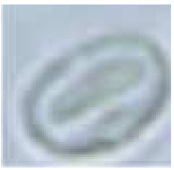

15

A que classe este pólen pertence?

- Alfazema-brava
- Almecega
- Angico
- Aroeira
- Arranha-gato
- Brachiaria
- Café-do-mato
- Camboatá
- Canela-de-ema
- Carvão-vermelho
- Cipó-neve
- Cipó-uva
- Dorme-dorme
- Eucalipto
- Guariroba
- Imbaúba
- Mamoeiro-selvagem
- Margarida-do-campo
- Mata-pasto
- Myrcia
- Palmeira
- Pau-terra
- Sangra d' água

16

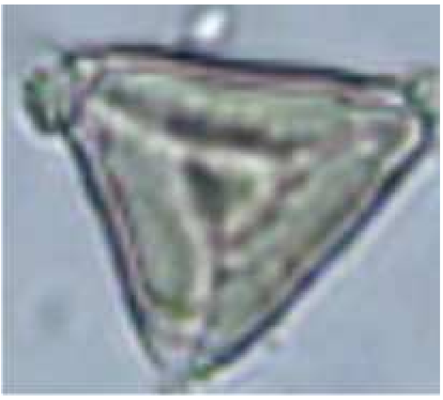

16

A que classe este pólen pertence?

- Alfazema-brava
- Almecega
- Angico
- Aroeira
- Arranha-gato
- Brachiaria
- Café-do-mato
- Camboatá
- Canela-de-ema
- Carvão-vermelho
- Cipó-neve
- Cipó-uva
- Dorme-dorme
- Eucalipto
- Guariroba
- Imbaúba
- Mamoeiro-selvagem
- Margarida-do-campo
- Mata-pasto
- Myrcia
- Palmeira
- Pau-terra
- Sangra d' água

17

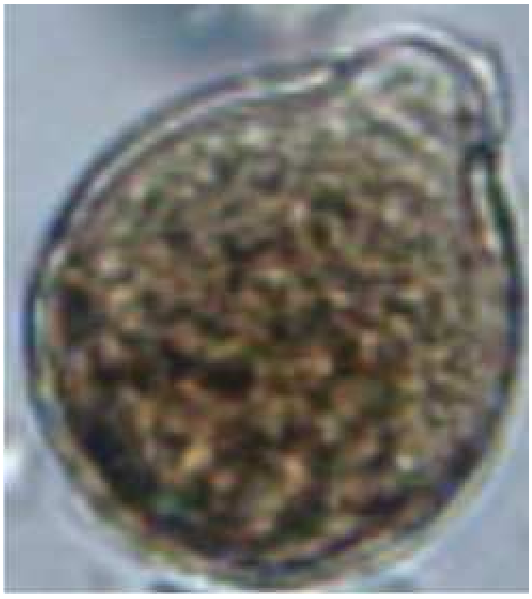

17

A que classe este pólen pertence?

- Alfazema-brava
- Almecega
- Angico
- Aroeira
- Arranha-gato
- Brachiaria
- Café-do-mato
- Camboatá
- Canela-de-ema
- Carvão-vermelho
- Cipó-neve
- Cipó-uva
- Dorme-dorme
- Eucalipto
- Guariroba
- Imbaúba
- Mamoeiro-selvagem
- Margarida-do-campo
- Mata-pasto
- Myrcia
- Palmeira
- Pau-terra
- Sangra d' água

18

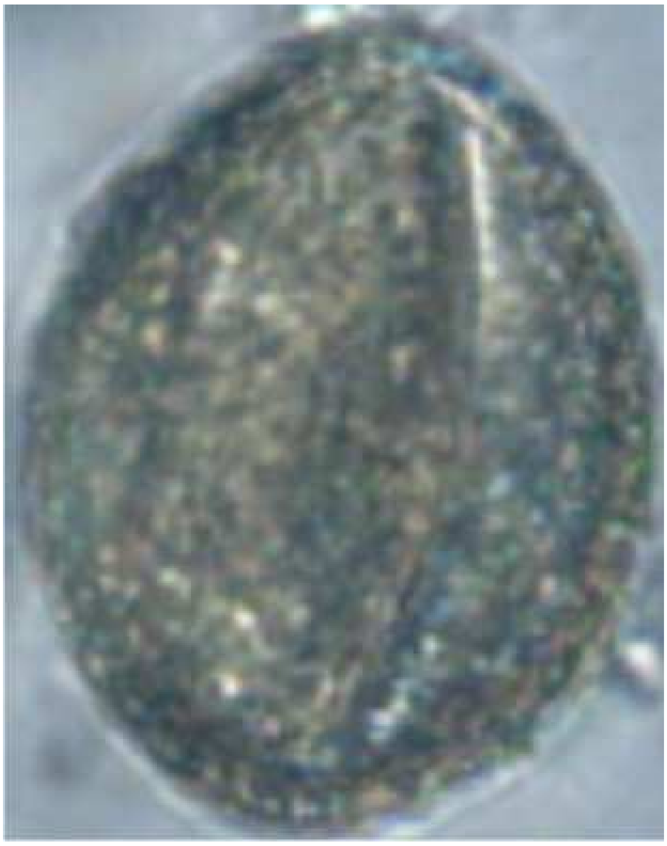

18

A que classe este pólen pertence?

- Alfazema-brava
- Almecega
- Angico
- Aroeira
- Arranha-gato
- Brachiaria
- Café-do-mato
- Camboatá
- Canela-de-ema
- Carvão-vermelho
- Cipó-neve
- Cipó-uva
- Dorme-dorme
- Eucalipto
- Guariroba
- Imbaúba
- Mamoeiro-selvagem
- Margarida-do-campo
- Mata-pasto
- Myrcia
- Palmeira
- Pau-terra

- Sangra d' água

19

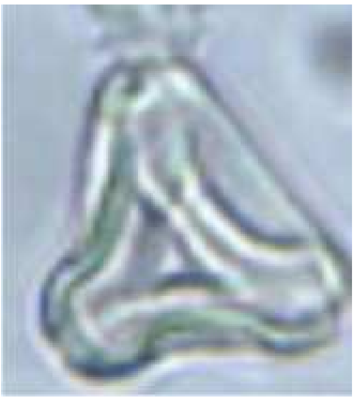

19

A que classe este pólen pertence?

- Alfazema-brava
- Almecega
- Angico
- Aroeira
- Arranha-gato
- Brachiaria
- Café-do-mato
- Camboatá
- Canela-de-ema
- Carvão-vermelho
- Cipó-neve
- Cipó-uva
- Dorme-dorme
- Eucalipto
- Guariroba
- Imbaúba
- Mamoeiro-selvagem
- Margarida-do-campo
- Mata-pasto
- Myrcia
- Palmeira
- Pau-terra
- Sangra d' água

20

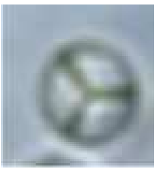

20

A que classe este pólen pertence?

- Alfazema-brava
- Almecega
- Angico
- Aroeira
- Arranha-gato
- Brachiaria
- Café-do-mato
- Camboatá
- Canela-de-ema
- Carvão-vermelho
- Cipó-neve
- Cipó-uva
- Dorme-dorme
- Eucalipto
- Guariroba
- Imbaúba
- Mamoeiro-selvagem
- Margarida-do-campo
- Mata-pasto
- Myrcia
- Palmeira
- Pau-terra
- Sangra d' água

21

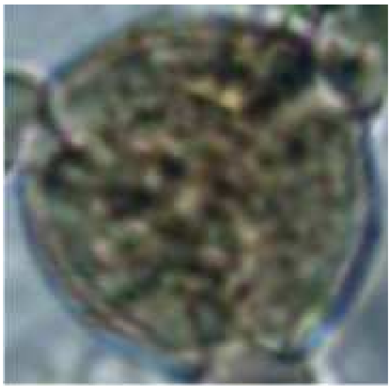

21

A que classe este pólen pertence?

- Alfazema-brava
- Almecega
- Angico
- Aroeira
- Arranha-gato
- Brachiaria
- Café-do-mato
- Camboatá
- Canela-de-ema
- Carvão-vermelho
- Cipó-neve
- Cipó-uva
- Dorme-dorme
- Eucalipto
- Guariroba
- Imbaúba
- Mamoeiro-selvagem
- Margarida-do-campo
- Mata-pasto
- Myrcia
- Palmeira
- Pau-terra
- Sangra d' água

22

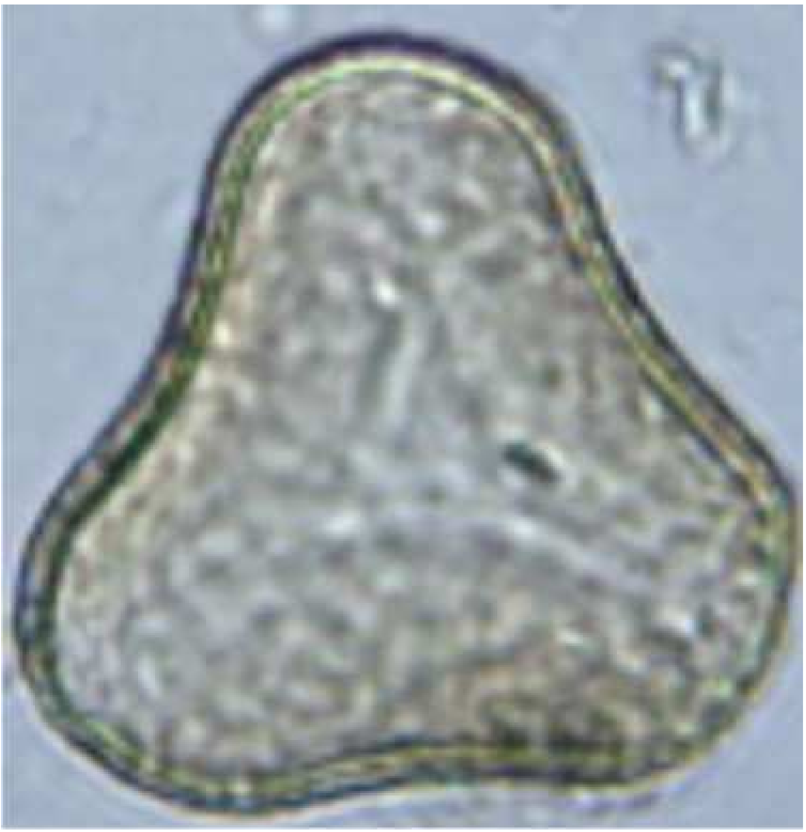

22

A que classe este pólen pertence?

- Alfazema-brava
- Almecega
- Angico
- Aroeira
- Arranha-gato
- Brachiaria
- Café-do-mato
- Camboatá
- Canela-de-ema
- Carvão-vermelho
- Cipó-neve
- Cipó-uva
- Dorme-dorme
- Eucalipto
- Guariroba
- Imbaúba
- Mamoeiro-selvagem
- Margarida-do-campo
- Mata-pasto
- Myrcia
- Palmeira
- Pau-terra
- Sangra d' água

23

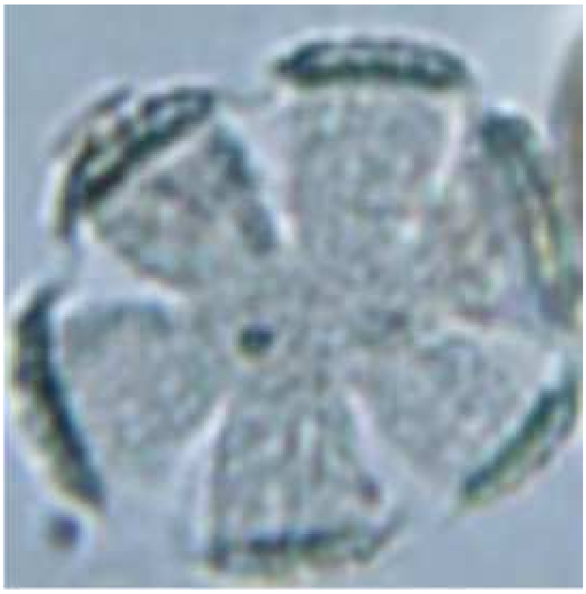

23

A que classe este pólen pertence?

- Alfazema-brava
- Almecega
- Angico
- Aroeira
- Arranha-gato
- Brachiaria
- Café-do-mato
- Camboatá
- Canela-de-ema
- Carvão-vermelho
- Cipó-neve
- Cipó-uva
- Dorme-dorme
- Eucalipto
- Guariroba
- Imbaúba
- Mamoeiro-selvagem
- Margarida-do-campo
- Mata-pasto
- Myrcia
- Palmeira
- Pau-terra
- Sangra d' água

24

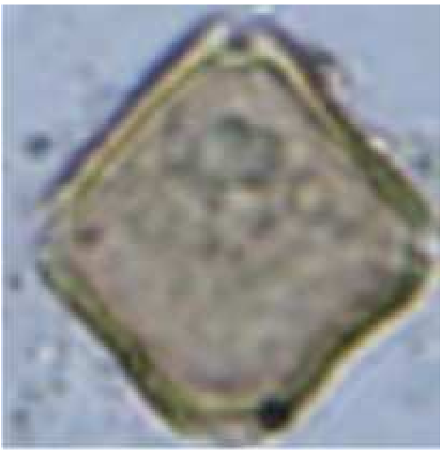

24

A que classe este pólen pertence?

- Alfazema-brava
- Almecega
- Angico
- Aroeira
- Arranha-gato
- Brachiaria
- Café-do-mato
- Camboatá
- Canela-de-ema
- Carvão-vermelho
- Cipó-neve
- Cipó-uva
- Dorme-dorme
- Eucalipto
- Guariroba
- Imbaúba
- Mamoeiro-selvagem
- Margarida-do-campo
- Mata-pasto
- Myrcia
- Palmeira
- Pau-terra
- Sangra d' água

25

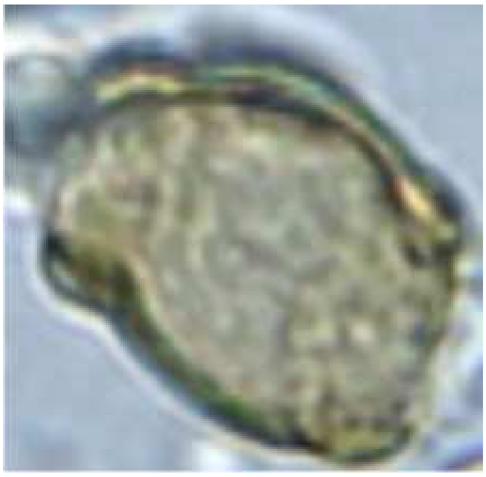

25

A que classe este pólen pertence?

- Alfazema-brava
- Almecega
- Angico
- Aroeira
- Arranha-gato
- Brachiaria
- Café-do-mato
- Camboatá
- Canela-de-ema
- Carvão-vermelho
- Cipó-neve
- Cipó-uva
- Dorme-dorme
- Eucalipto
- Guariroba
- Imbaúba
- Mamoeiro-selvagem
- Margarida-do-campo
- Mata-pasto
- Myrcia
- Palmeira
- Pau-terra
- Sangra d' água

26

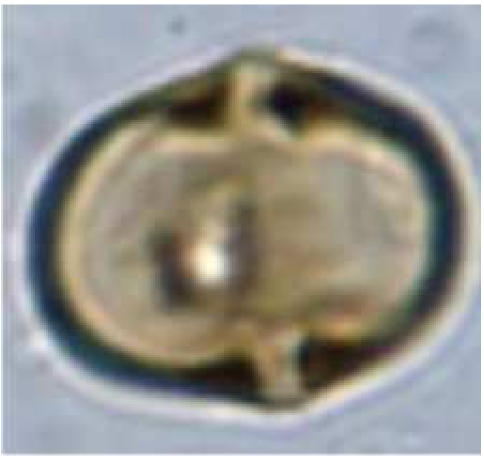

26

A que classe este pólen pertence?

- Alfazema-brava
- Almecega
- Angico
- Aroeira
- Arranha-gato
- Brachiaria
- Café-do-mato
- Camboatá
- Canela-de-ema
- Carvão-vermelho
- Cipó-neve
- Cipó-uva
- Dorme-dorme
- Eucalipto
- Guariroba
- Imbaúba
- Mamoeiro-selvagem
- Margarida-do-campo
- Mata-pasto
- Myrcia
- Palmeira
- Pau-terra
- Sangra d' água

27

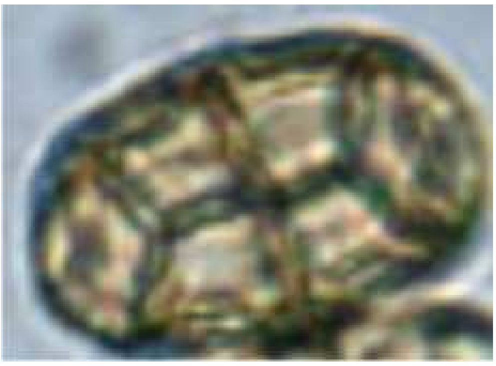

27

A que classe este pólen pertence?

- Alfazema-brava
- Almecega
- Angico
- Aroeira
- Arranha-gato
- Brachiaria
- Café-do-mato
- Camboatá
- Canela-de-ema
- Carvão-vermelho
- Cipó-neve
- Cipó-uva
- Dorme-dorme
- Eucalipto
- Guariroba
- Imbaúba
- Mamoeiro-selvagem
- Margarida-do-campo
- Mata-pasto
- Myrcia
- Palmeira
- Pau-terra
- Sangra d' água

28

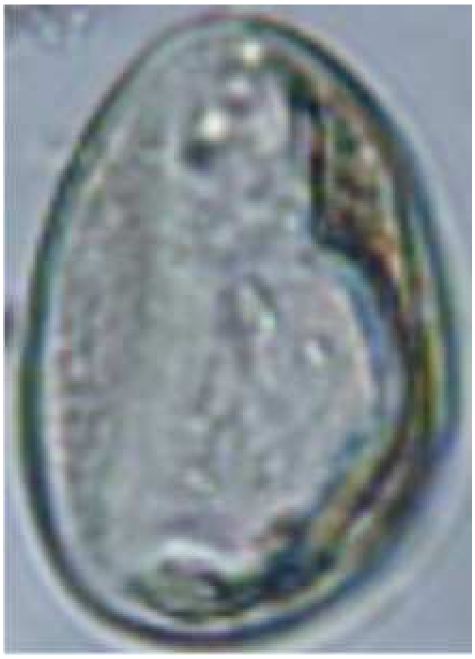

28

A que classe este pólen pertence?

- Alfazema-brava
- Almecega
- Angico
- Aroeira
- Arranha-gato
- Brachiaria
- Café-do-mato
- Camboatá
- Canela-de-ema
- Carvão-vermelho
- Cipó-neve
- Cipó-uva
- Dorme-dorme
- Eucalipto
- Guariroba
- Imbaúba
- Mamoeiro-selvagem
- Margarida-do-campo
- Mata-pasto
- Myrcia
- Palmeira
- Pau-terra
- Sangra d' água

29

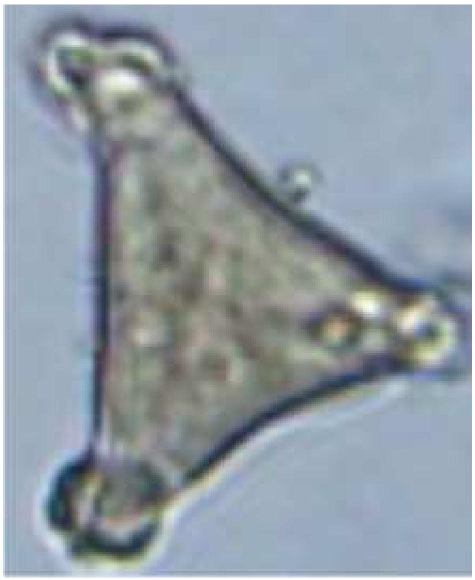

29

A que classe este pólen pertence?

- Alfazema-brava
- Almecega
- Angico
- Aroeira
- Arranha-gato
- Brachiaria
- Café-do-mato
- Camboatá
- Canela-de-ema
- Carvão-vermelho
- Cipó-neve
- Cipó-uva
- Dorme-dorme
- Eucalipto
- Guariroba
- Imbaúba
- Mamoeiro-selvagem
- Margarida-do-campo
- Mata-pasto
- Myrcia
- Palmeira
- Pau-terra
- Sangra d' água

30

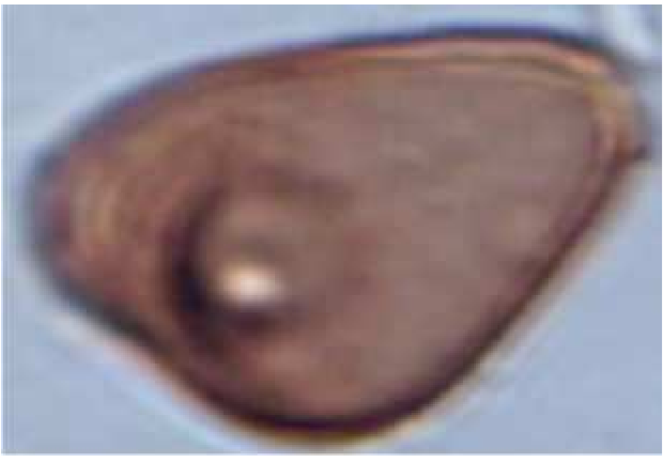

30

A que classe este pólen pertence?

- Alfazema-brava
- Almecega
- Angico
- Aroeira
- Arranha-gato
- Brachiaria
- Café-do-mato
- Camboatá
- Canela-de-ema
- Carvão-vermelho
- Cipó-neve
- Cipó-uva
- Dorme-dorme
- Eucalipto
- Guariroba
- Imbaúba
- Mamoeiro-selvagem
- Margarida-do-campo
- Mata-pasto
- Myrcia
- Palmeira
- Pau-terra
- Sangra d' água

31

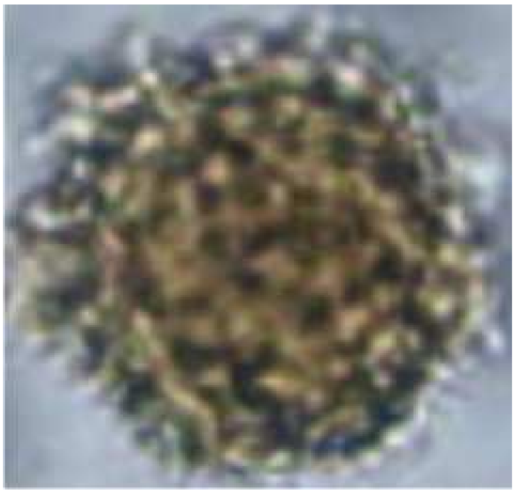

31

A que classe este pólen pertence?

- Alfazema-brava
- Almecega
- Angico
- Aroeira
- Arranha-gato
- Brachiaria
- Café-do-mato
- Camboatá
- Canela-de-ema
- Carvão-vermelho
- Cipó-neve
- Cipó-uva
- Dorme-dorme
- Eucalipto
- Guariroba
- Imbaúba
- Mamoeiro-selvagem
- Margarida-do-campo
- Mata-pasto
- Myrcia
- Palmeira
- Pau-terra
- Sangra d' água

32

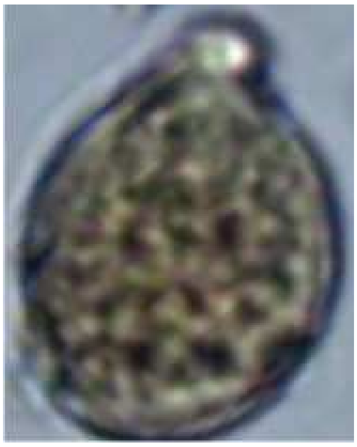

32

A que classe este pólen pertence?

- Alfazema-brava
- Almecega
- Angico
- Aroeira
- Arranha-gato
- Brachiaria
- Café-do-mato
- Camboatá
- Canela-de-ema
- Carvão-vermelho
- Cipó-neve
- Cipó-uva
- Dorme-dorme
- Eucalipto
- Guariroba
- Imbaúba
- Mamoeiro-selvagem
- Margarida-do-campo
- Mata-pasto
- Myrcia
- Palmeira
- Pau-terra
- Sangra d' água

33

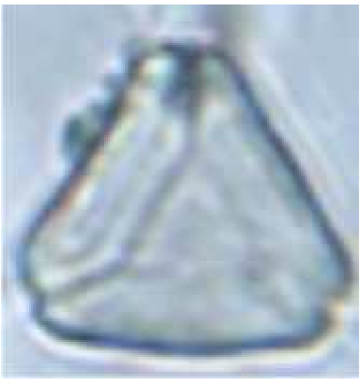

33

A que classe este pólen pertence?

- Alfazema-brava
- Almecega
- Angico
- Aroeira
- Arranha-gato
- Brachiaria
- Café-do-mato
- Camboatá
- Canela-de-ema
- Carvão-vermelho
- Cipó-neve
- Cipó-uva
- Dorme-dorme
- Eucalipto
- Guariroba
- Imbaúba
- Mamoeiro-selvagem
- Margarida-do-campo
- Mata-pasto
- Myrcia
- Palmeira
- Pau-terra
- Sangra d' água

34

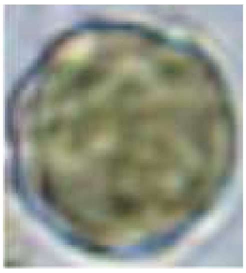

34

A que classe este pólen pertence?

- Alfazema-brava
- Almecega
- Angico
- Aroeira
- Arranha-gato
- Brachiaria
- Café-do-mato
- Camboatá
- Canela-de-ema
- Carvão-vermelho
- Cipó-neve
- Cipó-uva
- Dorme-dorme
- Eucalipto
- Guariroba
- Imbaúba
- Mamoeiro-selvagem
- Margarida-do-campo
- Mata-pasto
- Myrcia
- Palmeira
- Pau-terra
- Sangra d' água

35

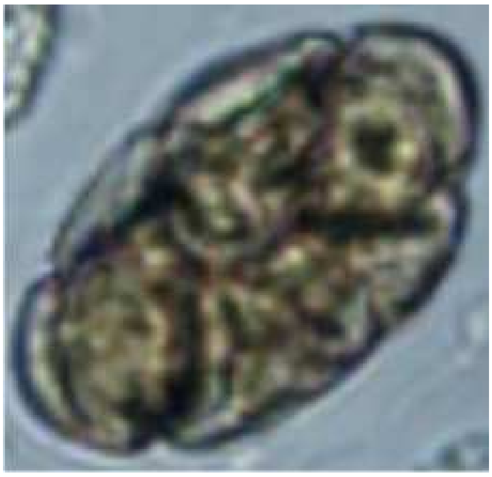

35

A que classe este pólen pertence?

- Alfazema-brava
- Almecega

- Angico
- Aroeira
- Arranha-gato
- Brachiaria
- Café-do-mato
- Camboatá
- Canela-de-ema
- Carvão-vermelho
- Cipó-neve
- Cipó-uva
- Dorme-dorme
- Eucalipto
- Guariroba
- Imbaúba
- Mamoeiro-selvagem
- Margarida-do-campo
- Mata-pasto
- Myrcia
- Palmeira
- Pau-terra
- Sangra d' água

36

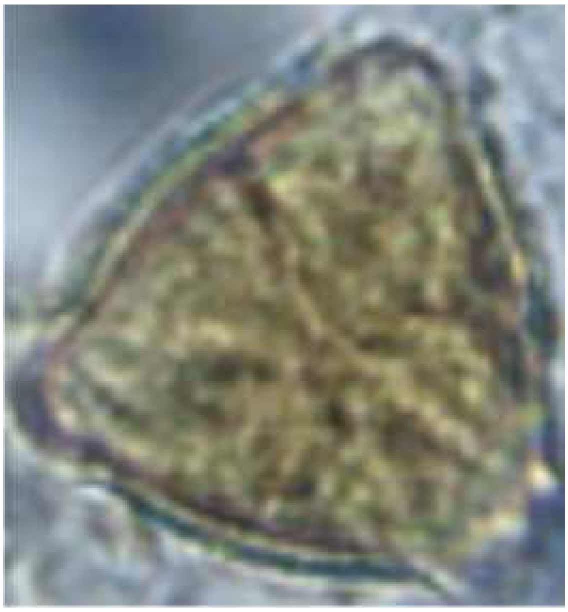

36

A que classe este pólen pertence?

- Alfazema-brava
- Almecega
- Angico

- Aroeira
- Arranha-gato
- Brachiaria
- Café-do-mato
- Camboatá
- Canela-de-ema
- Carvão-vermelho
- Cipó-neve
- Cipó-uva
- Dorme-dorme
- Eucalipto
- Guariroba
- Imbaúba
- Mamoeiro-selvagem
- Margarida-do-campo
- Mata-pasto
- Myrcia
- Palmeira
- Pau-terra
- Sangra d' água

37

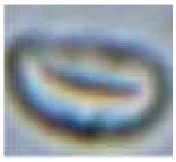

37

A que classe este pólen pertence?

- Alfazema-brava
- Almecega
- Angico
- Aroeira
- Arranha-gato
- Brachiaria
- Café-do-mato
- Camboatá
- Canela-de-ema
- Carvão-vermelho
- Cipó-neve
- Cipó-uva
- Dorme-dorme
- Eucalipto

- Guariroba
- Imbaúba
- Mamoeiro-selvagem
- Margarida-do-campo
- Mata-pasto
- Myrcia
- Palmeira
- Pau-terra
- Sangra d' água

38

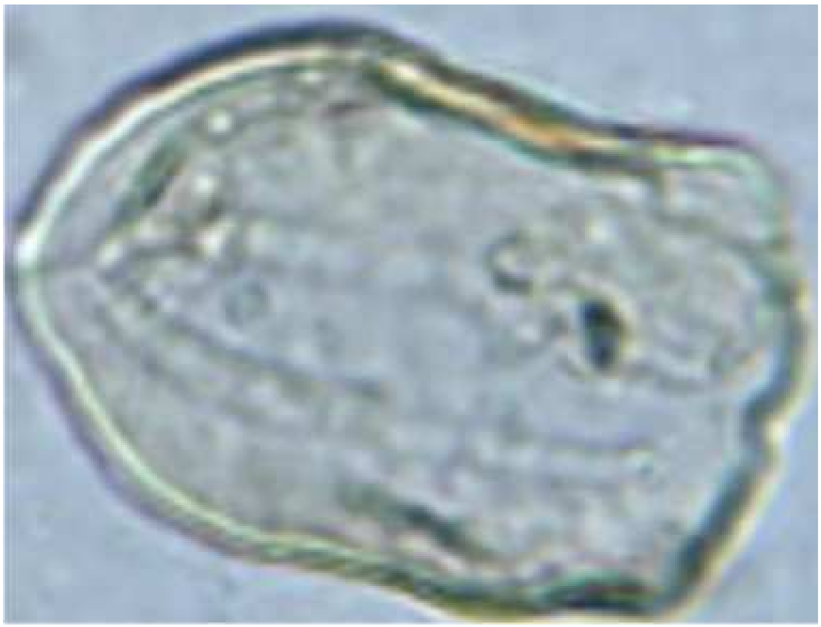

38

A que classe este pólen pertence?

- Alfazema-brava
- Almecega
- Angico
- Aroeira
- Arranha-gato
- Brachiaria
- Café-do-mato
- Camboatá
- Canela-de-ema
- Carvão-vermelho
- Cipó-neve
- Cipó-uva
- Dorme-dorme
- Eucalipto
- Guariroba

- Imbaúba
- Mamoeiro-selvagem
- Margarida-do-campo
- Mata-pasto
- Myrcia
- Palmeira
- Pau-terra
- Sangra d' água

39

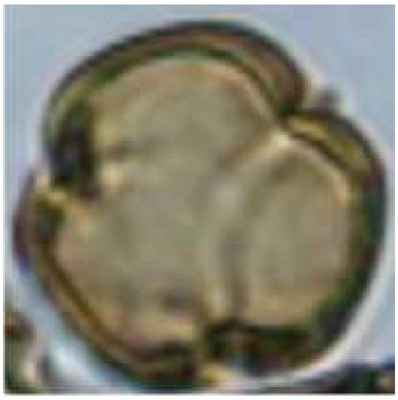

39

A que classe este pólen pertence?

- Alfazema-brava
- Almecega
- Angico
- Aroeira
- Arranha-gato
- Brachiaria
- Café-do-mato
- Camboatá
- Canela-de-ema
- Carvão-vermelho
- Cipó-neve
- Cipó-uva
- Dorme-dorme
- Eucalipto
- Guariroba
- Imbaúba
- Mamoeiro-selvagem
- Margarida-do-campo
- Mata-pasto
- Myrcia
- Palmeira

- Pau-terra
- Sangra d' água

40

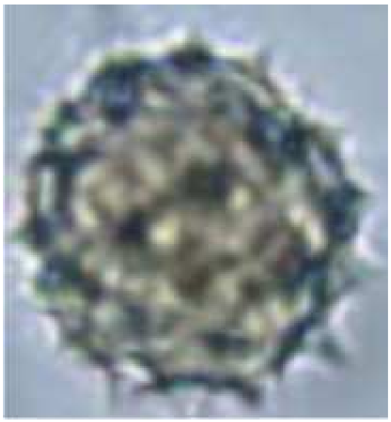

40

A que classe este pólen pertence?

- Alfazema-brava
- Almecega
- Angico
- Aroeira
- Arranha-gato
- Brachiaria
- Café-do-mato
- Camboatá
- Canela-de-ema
- Carvão-vermelho
- Cipó-neve
- Cipó-uva
- Dorme-dorme
- Eucalipto
- Guariroba
- Imbaúba
- Mamoeiro-selvagem
- Margarida-do-campo
- Mata-pasto
- Myrcia
- Palmeira
- Pau-terra
- Sangra d' água

41

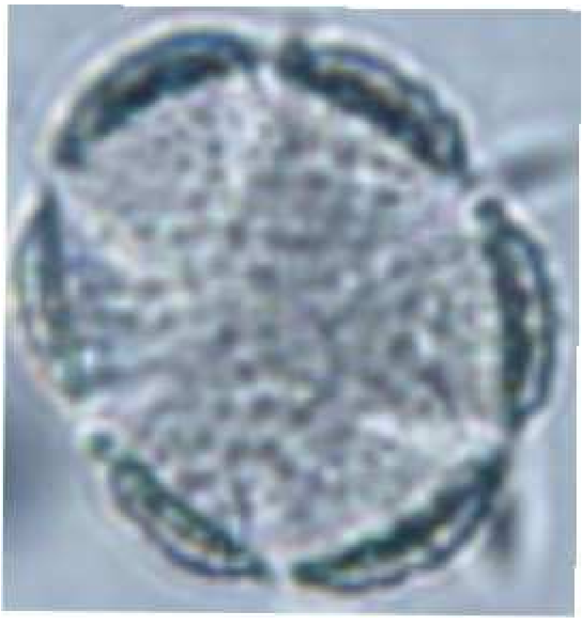

41

A que classe este pólen pertence?

- Alfazema-brava
- Almecega
- Angico
- Aroeira
- Arranha-gato
- Brachiaria
- Café-do-mato
- Camboatá
- Canela-de-ema
- Carvão-vermelho
- Cipó-neve
- Cipó-uva
- Dorme-dorme
- Eucalipto
- Guariroba
- Imbaúba
- Mamoeiro-selvagem
- Margarida-do-campo
- Mata-pasto
- Myrcia
- Palmeira
- Pau-terra
- Sangra d' água

42

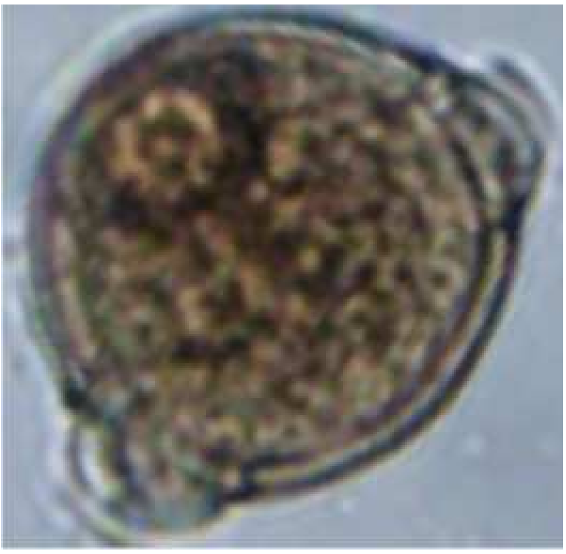

42

A que classe este pólen pertence?

- Alfazema-brava
- Almecega
- Angico
- Aroeira
- Arranha-gato
- Brachiaria
- Café-do-mato
- Camboatá
- Canela-de-ema
- Carvão-vermelho
- Cipó-neve
- Cipó-uva
- Dorme-dorme
- Eucalipto
- Guariroba
- Imbaúba
- Mamoeiro-selvagem
- Margarida-do-campo
- Mata-pasto
- Myrcia
- Palmeira
- Pau-terra
- Sangra d' água

43

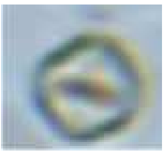

43

A que classe este pólen pertence?

- Alfazema-brava
- Almecega
- Angico
- Aroeira
- Arranha-gato
- Brachiaria
- Café-do-mato
- Camboatá
- Canela-de-ema
- Carvão-vermelho
- Cipó-neve
- Cipó-uva
- Dorme-dorme
- Eucalipto
- Guariroba
- Imbaúba
- Mamoeiro-selvagem
- Margarida-do-campo
- Mata-pasto
- Myrcia
- Palmeira
- Pau-terra
- Sangra d' água

44

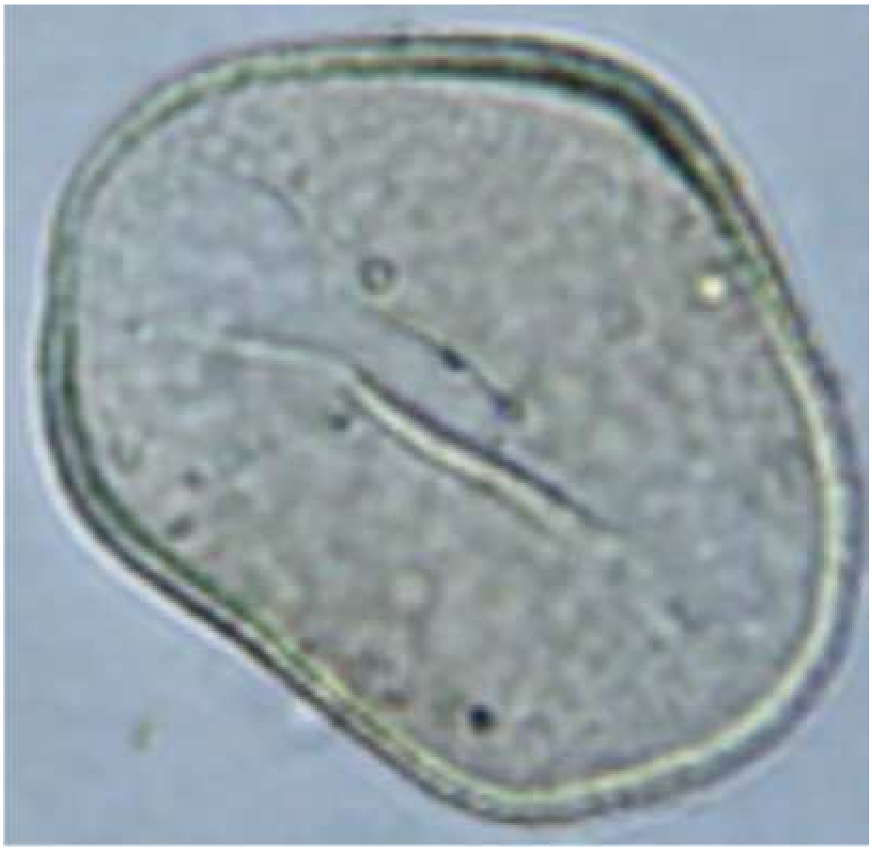

44

A que classe este pólen pertence?

- Alfazema-brava
- Almecega
- Angico
- Aroeira
- Arranha-gato
- Brachiaria
- Café-do-mato
- Camboatá
- Canela-de-ema
- Carvão-vermelho
- Cipó-neve
- Cipó-uva
- Dorme-dorme
- Eucalipto
- Guariroba
- Imbaúba
- Mamoeiro-selvagem
- Margarida-do-campo
- Mata-pasto
- Myrcia
- Palmeira
- Pau-terra

- Sangra d' água

45

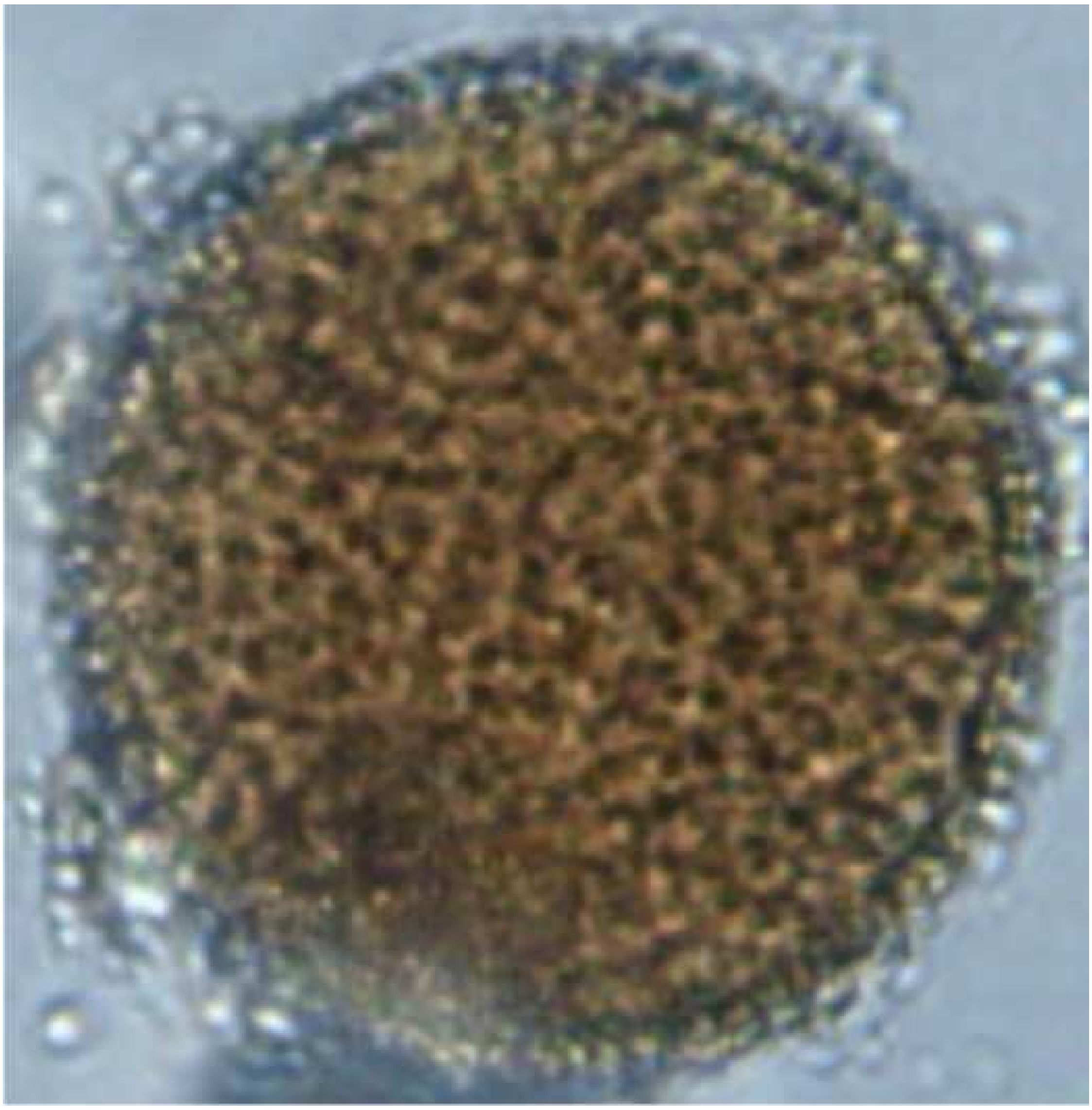

45

A que classe este pólen pertence?

- Alfazema-brava
- Almecega
- Angico
- Aroeira
- Arranha-gato
- Brachiaria
- Café-do-mato
- Camboatá
- Canela-de-ema
- Carvão-vermelho
- Cipó-neve
- Cipó-uva

- Dorme-dorme
- Eucalipto
- Guariroba
- Imbaúba
- Mamoeiro-selvagem
- Margarida-do-campo
- Mata-pasto
- Myrcia
- Palmeira
- Pau-terra
- Sangra d' água

46

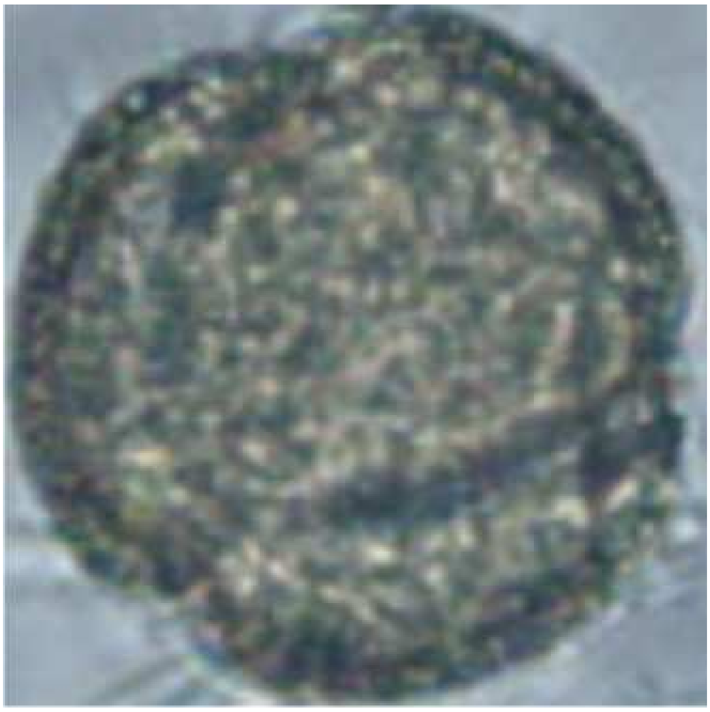

46

A que classe este pólen pertence?

- Alfazema-brava
- Almecega
- Angico
- Aroeira
- Arranha-gato
- Brachiaria
- Café-do-mato
- Camboatá
- Canela-de-ema
- Carvão-vermelho
- Cipó-neve

- Cipó-uva
- Dorme-dorme
- Eucalipto
- Guariroba
- Imbaúba
- Mamoeiro-selvagem
- Margarida-do-campo
- Mata-pasto
- Myrcia
- Palmeira
- Pau-terra
- Sangra d' água

47
Quais foram suas dificuldades na identificação dos polens?

48
Quais polens teve maior dificuldade para identificar e por quê?

49
O que observou na imagem para poder classificar o pólen?

Enviar

Nunca envie senhas em Formulários Google.
