# Supplementary material for: Feature Extraction and Machine Learning for the Classification of Brazilian Savannah Pollen Grains
Source: PLoS One. 2016 Jun 8;11(6):e0157044. doi: 10.1371/journal.pone.0157044 (PMC4898734; doi:10.1371/journal.pone.0157044)

MATERIAL DE APOIO

1. **ARRANHA-GATO**

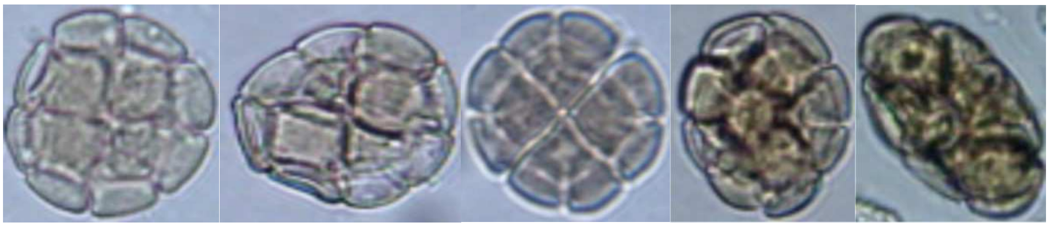

2. **ANGICO**

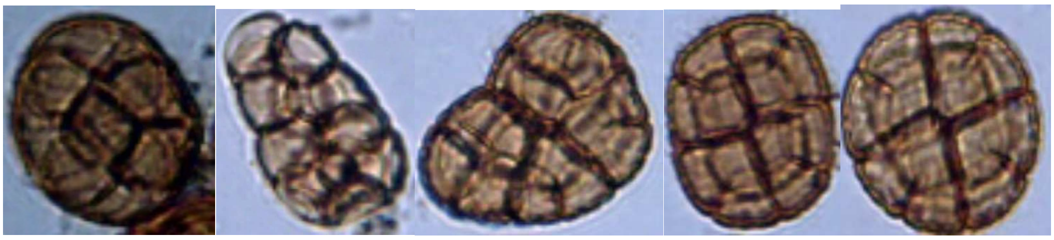

3. **PALMEIRA**

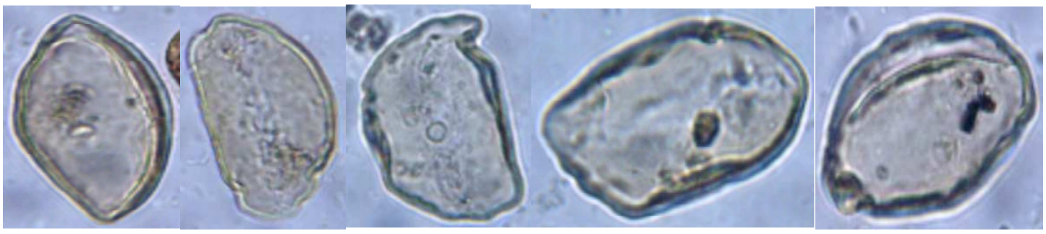

4. **GUARIROBA**

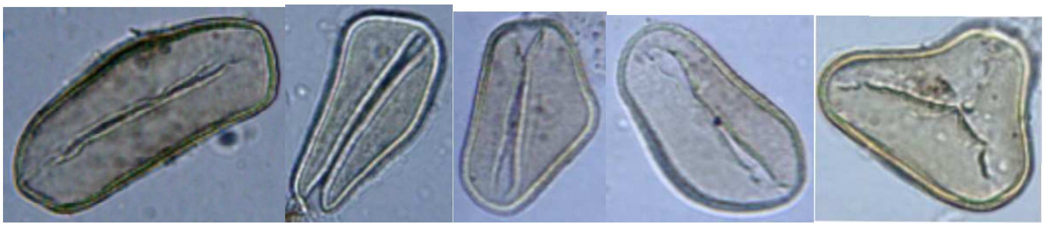

5. **CIPÓ-NEVE**

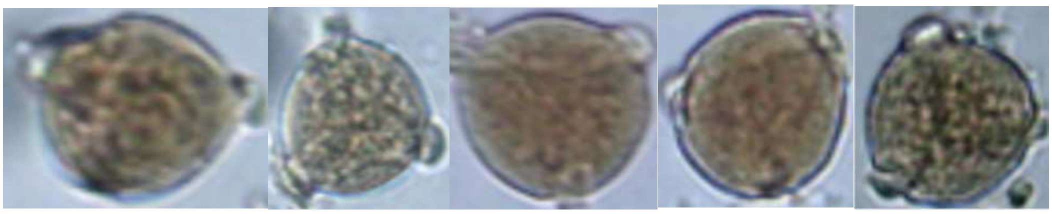

6. **CARVÃO-VERMELHO**

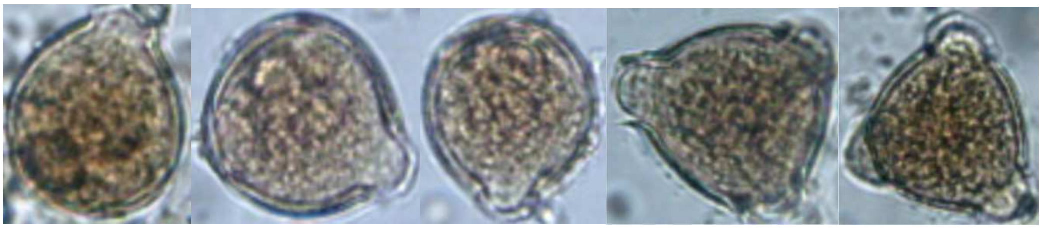

7. **PAU-TERRA**

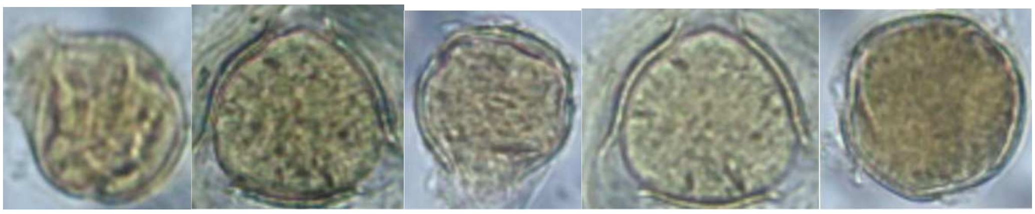

8. IMBAÚBA

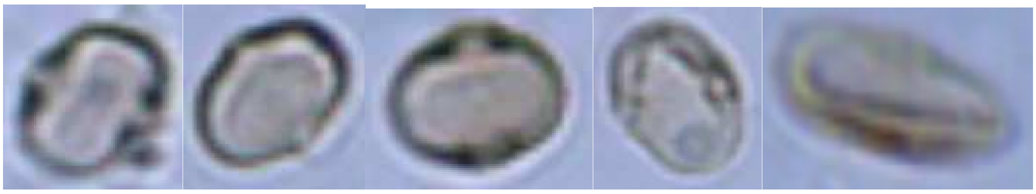

9. DORME-DORME

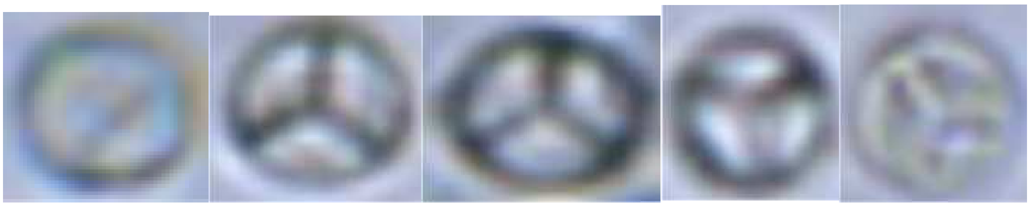

10. CAFÉ-DO-MATO

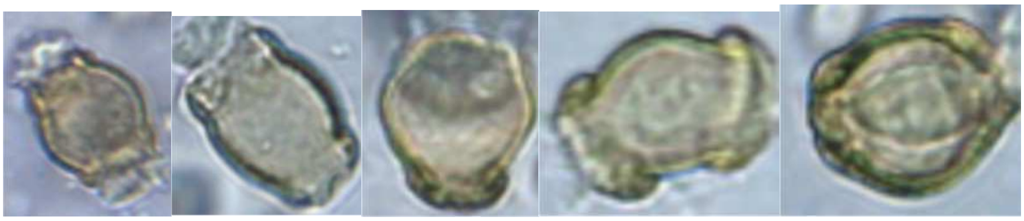

11. MATA-PASTO

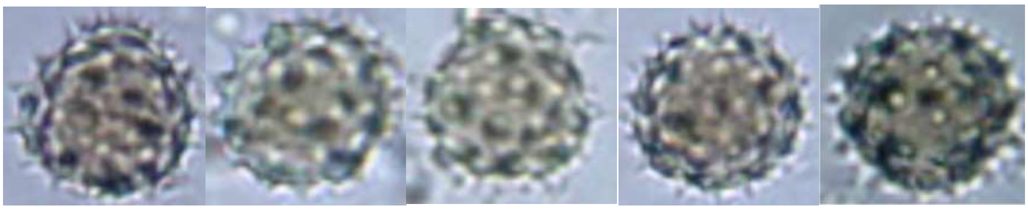

12. MARGARIDA-DO-CAMPO

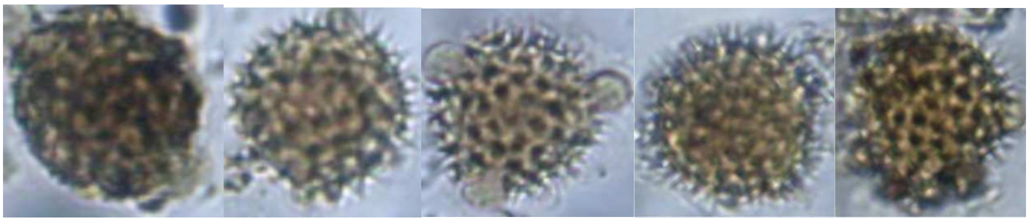

13. CANELA-DE-EMA

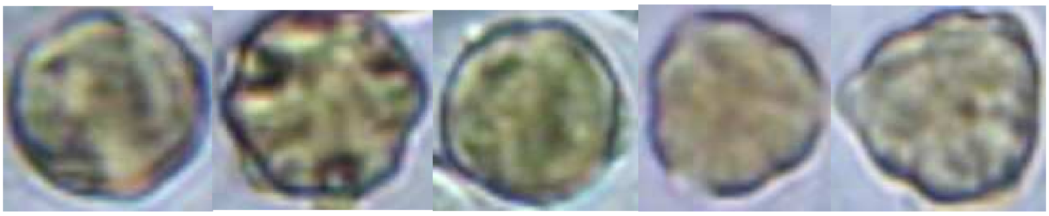

14. AROEIRA

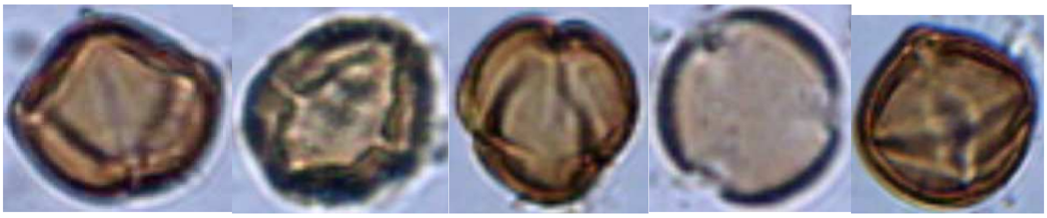

15. SANGRA D’ ÁGUA

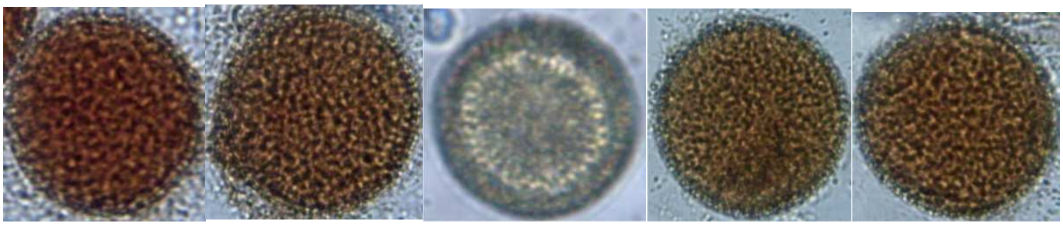

16. BRACHIARIA

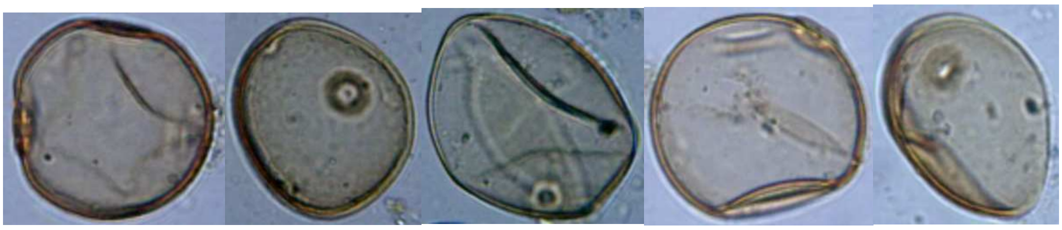

17. ALFAZEMA-BRAVA

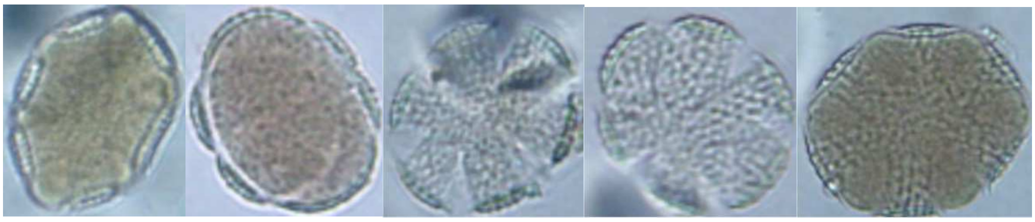

18. MAMOEIRO-SELVAGEM

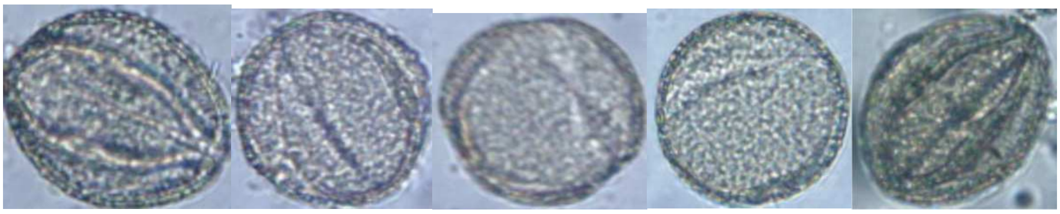

19. EUCALIPTO

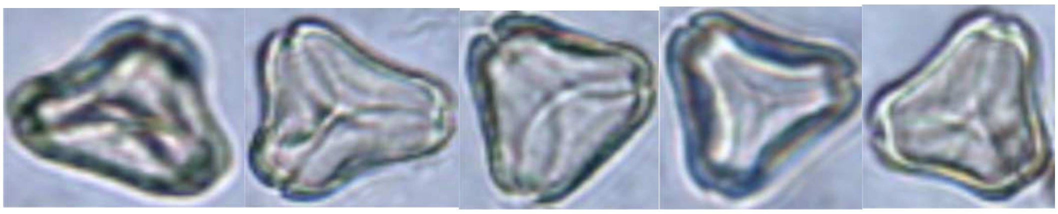

20. CAMBOATA

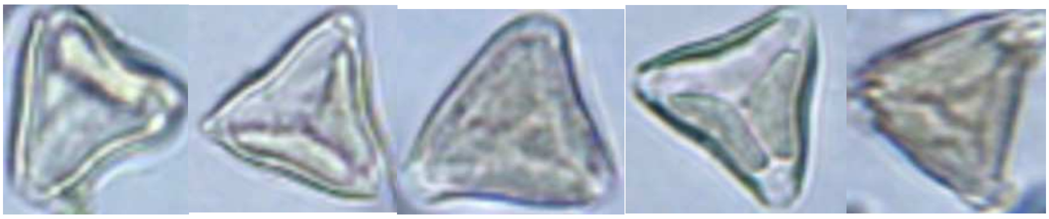

21. MYRCIA

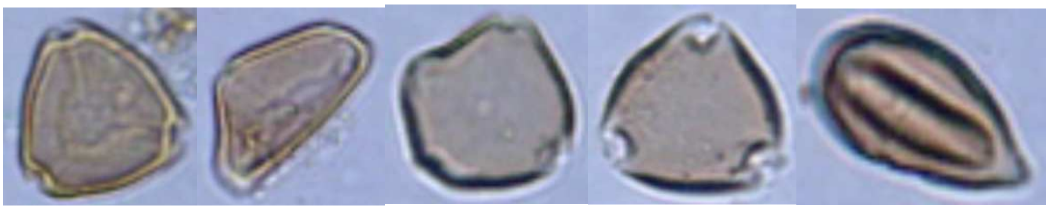

22. CIPÓ-UVA

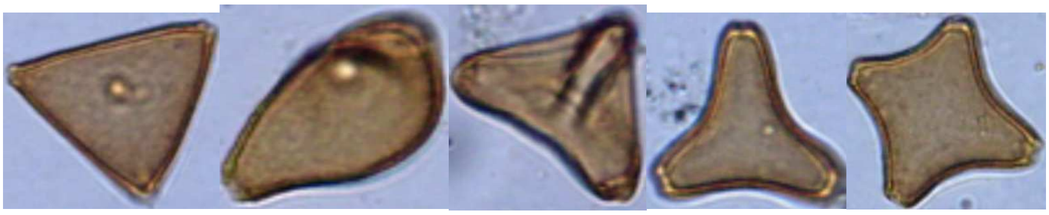

23. ALMECEGA

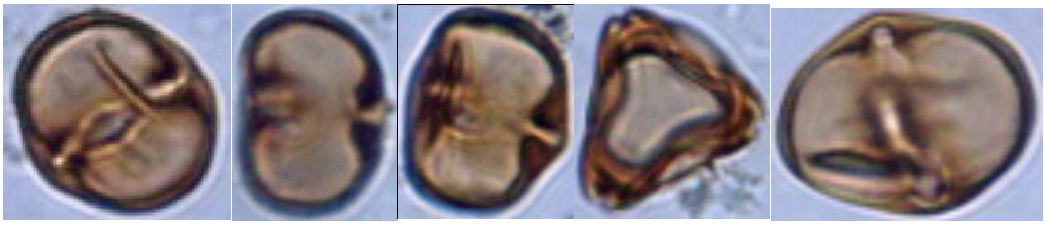

Supplement: S3 Text — (PDF) [file pone.0157044.s004.pdf]
